# Identification of Highly Selective Surface Pathways for Methane Dry Reforming Using Mechanochemical Synthesis of Pd–CeO_2_

**DOI:** 10.1021/acscatal.2c01120

**Published:** 2022-10-07

**Authors:** Juan D. Jiménez, Luis E. Betancourt, Maila Danielis, Hong Zhang, Feng Zhang, Ivan Orozco, Wenqian Xu, Jordi Llorca, Ping Liu, Alessandro Trovarelli, José A. Rodríguez, Sara Colussi, Sanjaya D. Senanayake

**Affiliations:** † Chemistry Division, Brookhaven National Laboratory, Upton, New York11793, United States; ‡ Polytechnic Department and INSTM, University of Udine, Via del Cotonificio 108, 33100Udine, Italy; § Department of Chemistry, State University of New York Stony Brook, Stony Brook, New York11794, United States; ∥ X-ray Science Division, Advanced Photon Source, Argonne National Laboratory, Lemont, Illinois60439, United States; ⊥ Department of Chemical Engineering, Institute of Energy Technologies, Universitat Politécnica de Catalunya, EEBE, Eduard Maristany 10-14, 08018Barcelona, Spain

**Keywords:** carbon dioxide, ceria, dry reforming, mechanochemistry, methane, palladium, isotopic labeling, reaction mechanism

## Abstract

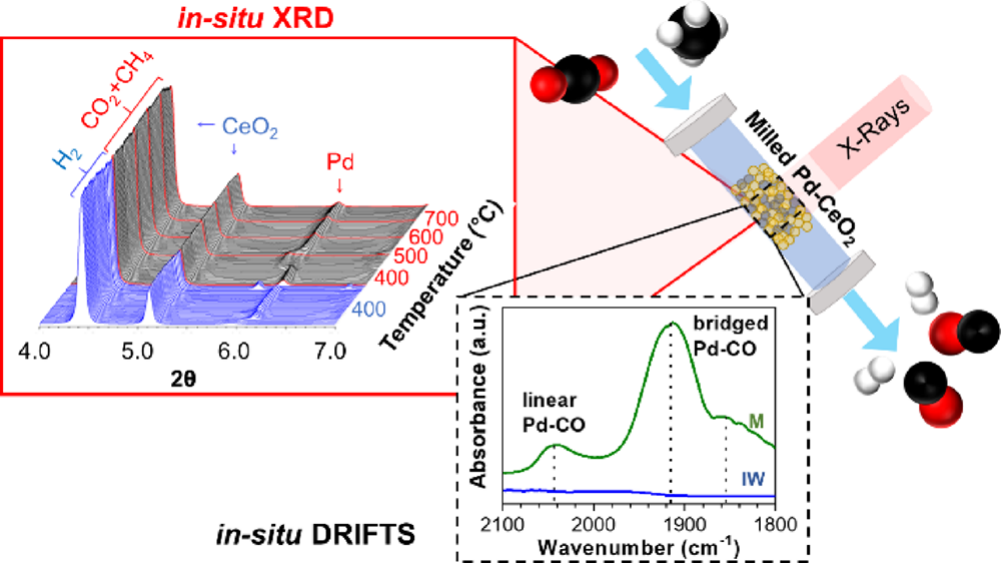

The methane dry reforming (DRM) reaction mechanism was explored via mechanochemically prepared Pd/CeO_2_ catalysts (PdAcCeO_2_M), which yield unique Pd–Ce interfaces, where PdAcCeO_2_M has a distinct reaction mechanism and higher reactivity for DRM relative to traditionally synthesized impregnated Pd/CeO_2_ (PdCeO_2_IW). In situ characterization and density functional theory calculations revealed that the enhanced chemistry of PdAcCeO_2_M can be attributed to the presence of a carbon-modified Pd^0^ and Ce^4+/3+^ surface arrangement, where distinct Pd–CO intermediate species and strong Pd–CeO_2_ interactions are activated and sustained exclusively under reaction conditions. This unique arrangement leads to highly selective and distinct surface reaction pathways that prefer the direct oxidation of CH_*x*_ to CO, identified on PdAcCeO_2_M using isotope labeled diffuse reflectance infrared Fourier transform spectroscopy and highlighting linear Pd–CO species bound on metallic and C-modified Pd, leading to adsorbed HCOO [1595 cm^–1^] species as key DRM intermediates, stemming from associative CO_2_ reduction. The milled materials contrast strikingly with surface processes observed on IW samples (PdCeO_2_IW) where the competing reverse water gas shift reaction predominates.

## Introduction

Natural gas is used on a larger scale than other traditional fuels such as oil, coal, or biomass in response to increasing energy demands from booming industrial development, the increase in world population, and higher life expectancy.^1^ Mitigating the environmental impacts of fuel processing is essential. The dry reforming of methane (DRM, CH_4_ + CO_2_ ⇋ 2H_2_ + 2CO) is a promising pathway for syngas (H_2_/CO) production at a ratio of almost unity. DRM becomes more relevant when coupled with the Fischer–Tropsch reaction, converting syngas into upgraded chemicals.^2,3^ DRM is an endothermic reaction (Δ*H*°_298K_ = 247 kJ/mol) that has a high energy barrier associated with the stable nature of methane (C–H bond energy: 104 kcal/mol) as well as with the activation of CO_2_, a poor source of O for the scission of C–H bonds. Thus, not only is the DRM reaction thermodynamically challenging, but it is also kinetically slow, requiring an active catalyst able to facilitate C–H and C–O bond cleavage.^4^ The anchoring of an active metal onto high-surface-area oxides represents a promising approach for preparing active DRM catalysts, creating a metal–support interface, and tuning the electronic and chemical properties of the metal catalyst.^5^ Among many oxide supports, CeO_2_ is a promising material with stable sites for the incorporation of Pd nanoparticles. Moreover, recent studies have highlighted its promotional effect in the DRM reaction.^6^ Ceria redox chemistry has been extensively evaluated, where several studies have identified the role of oxygen and hydrogen within the fluorite lattice.^7−10^ The barriers associated with the two slowest reaction steps to methane dissociation, CH_4_ → CH_3_ + H and CH → C + H, have been previously studied using density functional theory (DFT) methods on Pd substrates. Structure sensitivity of Pd was reported whereby lower coordination notably increased the order of reactivity: Pd_79_ > Pd_140_ > Pd(211) ≥ Pd(111).^11^ Pd-ceria contact promotes a similar low coordination arrangement, enhancing interfacial interactions and facilitating CH_4_ activation, making Pd/CeO_2_ suitable for DRM.^12,13^ Colussi et al. showed that the incorporation of Pd atoms into the CeO_2_ lattice via solution combustion synthesis outperforms Pd/CeO_2_ catalysts prepared by incipient wetness impregnation (IW) in methane oxidation and has remarkable resistance to steam deactivation.^14,15^ Furthermore, the degree of interaction between Pd and Ce can greatly influence the overall catalytic activity of the material.^16−18^ Thus, there is a continuous search for methods that enhance metal-oxide interactions and can be used in the preparation of more efficient catalysts, and among these, mechanochemical synthesis is receiving considerable attention.^19−22^ In particular, the milling of Pd with CeO_2_ is a novel and facile method to produce methane oxidation catalysts, forming an active Pd–Ce configuration at the nanoscale level.^12,23,24^ Pd–Ce-based materials prepared by mechanochemical synthesis were also explored for the partial oxidation of methane, which showed that milling Pd with either an acetate or nitrate precursor resulted in increased activity relative to the preparation via incipient wetness, because of improved dry and steam reforming reactivity occurring at higher temperature. The enhanced performance was attributed to the unique Pd–Ce configuration and increased dispersion offered via milling.^25^ This produces a specific surface arrangement where Pd moieties are embedded in the outer layer of ceria particles with unique interfacial Pd–O–Ce sites that are highly effective for methane oxidation, which serves as a basis for extending the study under milder oxidative conditions such as those encountered when CO_2_ is used in the presence of methane. Here, we describe a highly active Pd/CeO_2_ DRM catalyst prepared by mechanochemical milling, showing that its distinctive catalytic behavior and surface chemistry can only be traced applying in situ techniques to differentiate it from conventionally prepared Pd–CeO_2_ based catalysts with the same composition and nominal Pd loading. To the best of our knowledge, this is the first, most explicit instance where specific Pd active sites on ceria that are formed exclusively via a dry milling procedure and the resulting reaction intermediates are identified under reaction conditions with experimental and computational evidence correlated with the DRM mechanism and yield fundamental understanding of the CH_4_ conversion pathway in the presence of CO_2_.

## Experimental Section

### Catalyst Synthesis

A series of 4 wt % Pd/CeO_2_ samples were prepared by dry mechanochemical synthesis (M) and incipient wetness impregnation (IW). Commercial ceria with a high surface area was used as support oxide after calcination at 900 °C for 3 h in static air, with a final surface area of 25 m^2^/g. Milled samples were prepared following a mild milling synthesis reported elsewhere.^24^ Briefly, 84.4 mg of palladium acetate (Sigma-Aldrich, 99.9%) (PdAcCeO_2_M) or 46.0 mg of PdO nanoparticles (Sigma-Aldrich) (PdOCeO_2_M) were milled with 960 mg of CeO_2_ in a Fritsch Pulverisette 23 mini-mill operating at 15 Hz for 20 or 10 min, respectively. For the control, 1.0 g of ceria was milled in the same setup for 15 min (CeO_2_–M). The milling equipment (a 15 mL milling bowl and one 15 mm milling sphere) was composed of yttria-stabilized zirconia (YSZ), a material that offers great resistance to abrasion and prevents contamination by other metals. X-ray diffraction (XRD), X-ray photoelectron spectroscopy (XPS), and inductively coupled plasma-mass spectrometry (ICP-MS) elemental analyses confirmed that under the gentle milling conditions employed there is no zirconia contamination from the milling equipment. A reference Pd/CeO_2_ sample was prepared by incipient wetness impregnation (PdCeO_2_IW) using an appropriate amount of commercial palladium nitrate solution (Sigma-Aldrich, 99.999%) to reach a final Pd loading of 4 wt %. After complete wetting, the powders were dried at 100 °C overnight and then calcined at 900 °C for 3 h in static air to remove all residual nitrates. Conversely, no additional treatments in air were performed on samples prepared by milling, following optimized procedures investigated elsewhere.^26^ However, on all samples the consolidation of the supported Pd species and the complete removal of residual nitrates and carbonaceous species were thoroughly followed during and after H_2_ pretreatment, as described in detail in the Catalytic Activity Tests and Catalyst Characterization sections. To verify the actual palladium loading of the prepared samples, ICP-MS elemental analysis was performed by Mikroanalytisches Labor Pascher (Remagen, Germany); on both catalysts, the measured Pd loading corresponded to 3.9 wt %.

### Catalytic Activity Tests

The catalytic performance for the DRM of the Pd–CeO_2_ catalysts after H_2_ reduction treatment at 400 °C was evaluated in the temperature range of 400–700 °C under a space velocity of 180,000 mL/(g_cat_·h). Activity tests for the DRM reaction were performed on the as-prepared samples. The powder catalysts (10.0 mg, 60–80 mesh) were diluted by ∼20 mg of precalcined quartz (900 °C, 60–80 mesh), loaded into a quartz tube, and mounted on a bed-flow system. The gas ratio of CH_4_ and CO_2_ was set at 1:1 (10 mL/min CH_4_ with 10 mL/min CO_2_) in the catalytic performance test diluted by N_2_ (10 mL/min). Prior to the reaction, the catalysts were reduced under a H_2_ flow (10 mL/min) at 400 °C for 1 h. Their DRM activity was measured at 400, 500, 600, and 700 °C, with one isothermal hour step at each corresponding temperature. The concentrations of gas products were analyzed with gas chromatography (Agilent 7890A) equipped with both flame ionization and thermal conductivity detectors. Conversion of reactants was calculated according to the following equation:
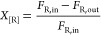
where *X* is the conversion, R indicates CH_4_ or CO_2,_ and *F* is the reactant flowrate (in or out).

The internal heat and mass transfer limitations of the system were explored via the Anderson and Weisz–Prater criteria, respectively, and were excluded under these working conditions (see the Supplementary Information).

### Catalyst Characterization

#### In Situ Time-Resolved XRD

Time-resolved XRD measurements were performed at beamline 17-BM (λ = 0.24169 Å) at the Advanced Photon Source (APS) using a Clausen cell flow reactor.^53^ A 10 mL/min flow rate of pure H_2_ was first used to pretreat the catalyst at 400 °C for 1 h. The gas line was subsequently purged with helium at room temperature before introducing a 10 mL/min flow of a gas mixture containing 20% CO_2_, 20% CH_4_, and 60% He for a 1:1 CO_2_/CH_4_ molar ratio. The samples were stepwise heated to 700 °C with a 10 °C/min ramping rate. An in-line residual gas analyzer was used to track the evolution of the gaseous species right after the flow cell. A Si flat detector (PerkinElmer) was used to collect two-dimensional XRD patterns throughout the reaction processes. The XRD data were subsequently processed using GSAS-II software.

#### In Situ X-ray Absorption Fine Structure

XAFS measurements of Pd–CeO_2_ catalysts were performed at 7-BM (QAS) beamline at National Synchrotron Light Source II (NSLS-II), Brookhaven National Laboratory (BNL). For the CH_4_-TPR experiment, the temperature of the catalyst inside the Clausen cell was ramped to 700 °C at 10 °C/min under 5 mL/min flow of CH_4_ diluted in a 15 mL/min of He. For the methane dry reforming experiment, the temperature was ramped in a manner similar to the in situ XRD measurements previously described. The Pd K-edge data were collected in fluorescence yield mode using a passivated implanted planar silicon detector. Data processing was performed using the IFEFFIT package. Pd foil was used as a standard reference for EXAFS fitting.

#### Ambient-Pressure X-ray Photoelectron Spectroscopy (AP-XPS)

A commercial SPECS AP-XPS chamber equipped with a PHOIBOS 150 EP MCD-9 analyzer at the Chemistry Division of BNL was used for XPS analysis.^5^ The Ce 3d photoemission line with the strongest Ce^4+^ feature (916.9 eV) was used for energy calibration. The powder catalyst was pressed on an aluminum plate and then loaded into the AP-XPS chamber. For CH_4_ TPR, 40 mTorr of O_2_ was used to pretreat the sample at 400 °C for 1 h before adding 10 mTorr of CH_4_ into the reaction chamber through a high-precision leak valve; for DRM, a mixture of 10 mTorr of CH_4_ and 10 mTorr of CO_2_ was introduced after H_2_ pretreatment (10 mTorr). O 1s, Ce 3d, and C 1s + Pd 3d XPS regions were collected from 25 to 500 °C under the reaction gas environment.

#### Transmission Electron Microscopy (TEM)

TEM was used to evaluate the morphological evolution of catalysts before (after H_2_ pretreatment), during (3 h stability experiments), and after reaction (24 h stability experiment). High-resolution TEM (HRTEM) together with high-angle annular dark-field (HAADF) scanning TEM (STEM) investigation was performed on a field emission gun FEI Tecnai F20 microscope at 200 kV.

### Diffuse Reflectance Infrared Fourier Transform Spectroscopy (DRIFTS)

In situ DRIFTS spectra were collected in Kubelka–Munk (K–M) mode using an FTIR spectrometer (Bruker Vertex 70) equipped with a Harrick Praying Mantis cell, an MCT detector, and mass spectroscopy. The catalyst was reduced in H_2_ (5 mL/min) at 400 °C for 1 h and then purged with 10 mL/min of He. The background was collected at 250 °C under He before introduction of gas reactants (CH_4_/CO_2_/He, 5/5/30 mL/min). For the “gas-on, gas-off” experiments, the gas flow was always balanced by helium to a total amount of 40 mL/min. Isotopic switching experiments were used to corroborate the degree of reaction intermediates among surface chemical species observed in DRIFTS. The catalyst sample was introduced to the DRIFTS cell and heated to 400 °C under 50% H_2_ and He (total 40 mL/min) for 1 h. Afterward, the DRIFTS cell was cooled to 250 °C under DRM reactant gas flow containing ^12^CO_2_. CO adsorption experiments were performed at the indicated temperature using a CO dosing of 10% CO/He (5/45 mL/min) for 15 min followed by 5 min of pure He (50 mL/min) purging for the CO desorption. The background spectra for CO adsorption experiments were collected under pure He flow (50 mL/min) at the indicated temperature, where the background was allowed to stabilize for three consecutive scans before data collection. Post DRM CO adsorption was first reduced in 10% H_2_/He (5/45 mL/min) at 400 °C for 1 h and then purged with 10 mL/min of He and then brought to room temperature; afterward, the catalysts were ramped up to 400 °C using a 10 °C/min ramp rate under DRM conditions (CH_4_/CO_2_/He, 5/5/30 mL/min) and held at temperature for 1 h. Post CH_4_ exposure CO adsorption had the same reductive treatment; however, the catalyst was ramped up to 400 °C under 25% CH_4_/He (12.5/37.5 mL/min) with a ramp rate of 10 °C/min and held at 400 °C for 5 min to stabilize the temperature; prolonged elevated temperature exposure under CH_4_ results in excessive carburization of the catalysts and a decreased IR signal; therefore, the sample was only held for 5 min under CH_4_ at 400 °C. All DRIFTS experiments had a spectral resolution of 4 cm^–1^.

### Theoretical Approach

The calculations were performed using the Vienna ab initio simulation package.^27−29^ The Perdew–Burke–Ernzerhof^30^ functional was used to describe electronic exchange and correlation. The pseudopotential files were prepared by Vaspkit.^31^ A 7 × 7 × 1 Monkhorst–Pack grid and first-order Methfessel–Paxton with a smearing width of 0.2 eV were used to integrate over the Brillouin zone. To describe the supported Pd catalysts, the closed-packed Pd(111) surface was considered. A 2 × 2 periodic slab unit cell with a four-layer thickness was constructed based on a *Fm*3̅*m* Pd unit cell with a lattice constant of 3.952 Å, consistent with experimental values.^32,33^ The bottom two layers were constrained as the bulk Pd FCC (face center cubic) unit, and a minimum 1.5 nm of vacuum spacing was placed between the slabs, while the rest was allowed to relax with CO. All systems were relaxed by the RMM-DIIS ionic relaxation algorithm^34^ with a plane wave cutoff energy of 400 eV to the level of 1 × 10^–6^ eV to obtain the convergence of electronic structure. The ionic relaxation was activated, and the atomic coordinates were updated until the Hellman–Feynman force was less than 0.02 eV/Å on each ion. The initial wavefunctions were calculated from scratch and took superposition of atomic charge densities, and all systems were dipole-corrected along the direction perpendicular to the Pd(111) surface. The binding energy (B.E.) of CO was calculated as B.E.(CO) = *E*_CO/Surface_ – *E*_Surface_ – *E*_CO_, where *E*_CO/Surface_, *E*_Surface_, and *E*_CO_ represent the total system energy of the CO-adsorbed surface, the bare surface, and the CO in gas phase, respectively.

## Results and Discussion

### Catalytic Evaluation of DRM for PdAcCeO_2_M and PdCeO_2_IW

**Figure 1:**
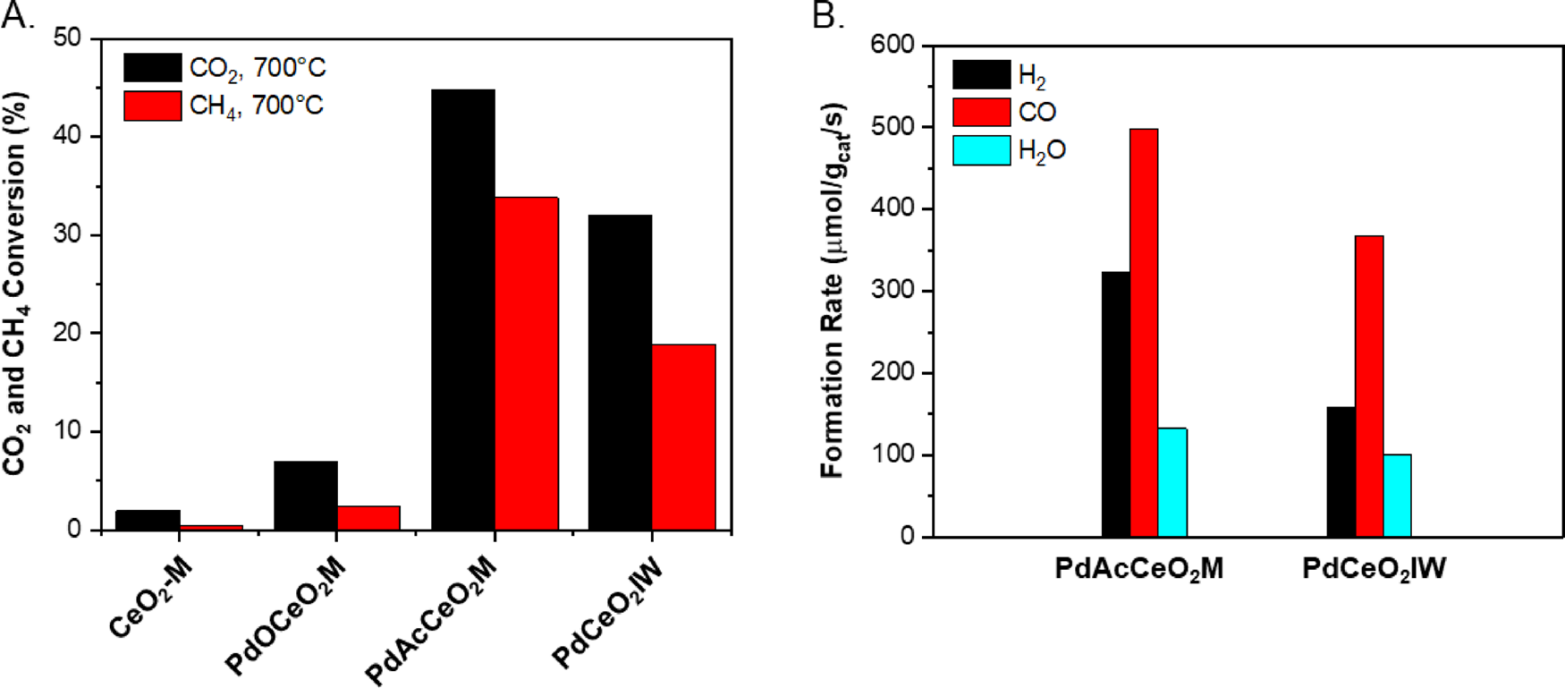
(A) CO_2_ and CH_4_ conversion measured under DRM conditions at 700 °C on 4 wt % Pd/CeO_2_ samples and bare CeO_2_–M support. (B) Reaction rates of H_2_, CO and H_2_O production during DRM at 700 °C. Reaction conditions: 10 mL/min CO_2_ + 10 mL/min CH_4_ + 10 mL/min N_2_ with 10.0 mg of catalyst; weight hourly space velocity (WHSV): 180,000 mL/(g_cat_·h).

As shown in Figure 1A, at 700 °C, the milled PdAcCeO_2_M sample exhibits a higher CH_4_ conversion (33.8%) than PdCeO_2_IW (18.9%), while PdOCeO_2_M is barely active and the Pd free milled ceria CeO_2_–M support shows negligible activity under the same reaction conditions. Conversion of CO_2_ is also higher for the milled catalyst: 44.8% compared to 32.1% in the impregnated sample. The deposition of Pd acetate onto ceria by milling leads to higher activity compared to just milling PdO onto ceria; therefore, milling by itself is not sufficient to create an active Pd–CeO_2_ arrangement.^24,26^ From selectivity results (Figure 1B), it can be observed that at 700 °C, PdAcCeO_2_M exhibited higher H_2_ production (323 μmol/g_cat_/s) compared to the impregnated catalyst (158.3 μmol/g_cat_/s), along with an increased CO formation rate, a higher H_2_/CO ratio, and a lower H_2_O production rate per mole of CH_4_ converted. Differences in rates of product formation might be ascribed to different side reactions taking place on the two samples. In general, the deposition of Pd nanoparticles on the surface of CeO_2_ creates oxygen vacancies leading to stronger anchoring sites for Pd entities while simultaneously increasing Pd dispersion.^12^ When methane interacts with Pd nanoparticles, it dissociates into hydrogen and carbon, which is subsequently oxidized by the oxygen species provided by CeO_2_ in close contact with palladium, producing CO as a main product. Alternatively, the carbon may be oxidized by dissociative adsorption of CO_2_, thus initiating the DRM reaction. At elevated temperatures, the production of hydrogen by methane dissociation leads to a reducing environment, facilitating CO_2_ dissociation and increasing CO_2_ conversion. The H_2_/CO ratio can be affected by the competing reverse water gas shift (RWGS, H_2_ + CO_2_ → H_2_O + CO) reaction, which is more prominent on PdCeO_2_IW with a higher water formation rate per mole of CH_4_ converted and is consistent with its lower selectivity to H_2_ production (Figure 1B). On the other hand, PdAcCeO_2_M shows a higher conversion for CO_2_, possibly ascribed to the simultaneous removal of deposited carbon during its reaction with CeO_2_ surface oxygen species (C + O → CO) and CO_2_ decomposition (CO_2_ + C ⇋ 2CO). The catalytic performance of both PdAcCeO_2_M and PdCeO_2_IW was also evaluated between 400 and 700 °C, where the milled catalyst had a consistently higher H_2_/CO ratio and less produced water per mole of CH_4_ converted (Figure S1). Furthermore, the reactivity of the single RWGS side reaction was evaluated between 400 and 700 °C, where both catalysts performed comparably for RWGS in term of both CO_2_ conversion and CO selectivity (>95% selectivity toward CO), showing the unique differences between PdAcCeO_2_M and PdCeO_2_IW are due to complex interaction during the DRM reaction and not a consequence of the independent side reactions (Figure S2), where PdCeO_2_IW shows more water and less H_2_ during DRM per mole of CH_4_ converted, suggesting that under DRM conditions PdCeO_2_IW promotes the RWGS.

Stability tests were performed by running an isothermal step at 700 °C for 24 h. The conversion profiles (Figure S3) under prolonged exposure to DRM conditions show that the milled catalyst was able to retain its initial activity with a sustained H_2_/CO ratio of 0.65, whereas the impregnated sample exhibited a 10% reduction in both CO_2_ and CH_4_ conversion with a H_2_/CO ratio of 0.43. In agreement with the activity measurements reported in Figure 1, PdAcCeO_2_M shows a higher CO_2_ conversion, with remarkable stability up to 40 h (Figure S4). In general, both PdAcCeO_2_M and PdCeO_2_IW show comparable relative DRM activity with various state-of-the-art catalysts (Table S1). The comparison though is only indicative, as each system has been tested under different operating conditions.

### Structural Properties of PdAcCeO_2_M and PdCeO_2_IW

HRTEM images of PdAcCeO_2_M after hydrogen reduction at 400 °C are reported in Figure S5(A,B) where palladium is well dispersed and partially embedded within the ceria lattice. The support exhibits lattice fringes at 3.1 Å corresponding to the (111) crystallographic planes of the ceria particles, while Pd nanoparticles exhibit lattice fringes at 2.2 and 1.9 Å corresponding to the (111) and (200) crystallographic planes of Pd metal, respectively. Pd nanoparticles exhibit an average particle size of ∼3.5 nm and no amorphous Pd–Ce–O shell is distinguishable, different from a previously reported Pd–CeO_2_ milled catalyst after treatment under oxidative conditions.^24^ HRTEM images of the nanostructures obtained by incipient wetness impregnation (PdCeO_2_IW) after hydrogen reduction are shown in Figure S5(C,D). These Pd metal particles are homogeneous in size (∼2 nm), slightly smaller than Pd nanoparticles on PdAcCeO_2_M and very well dispersed on ceria.

**Figure 2:**
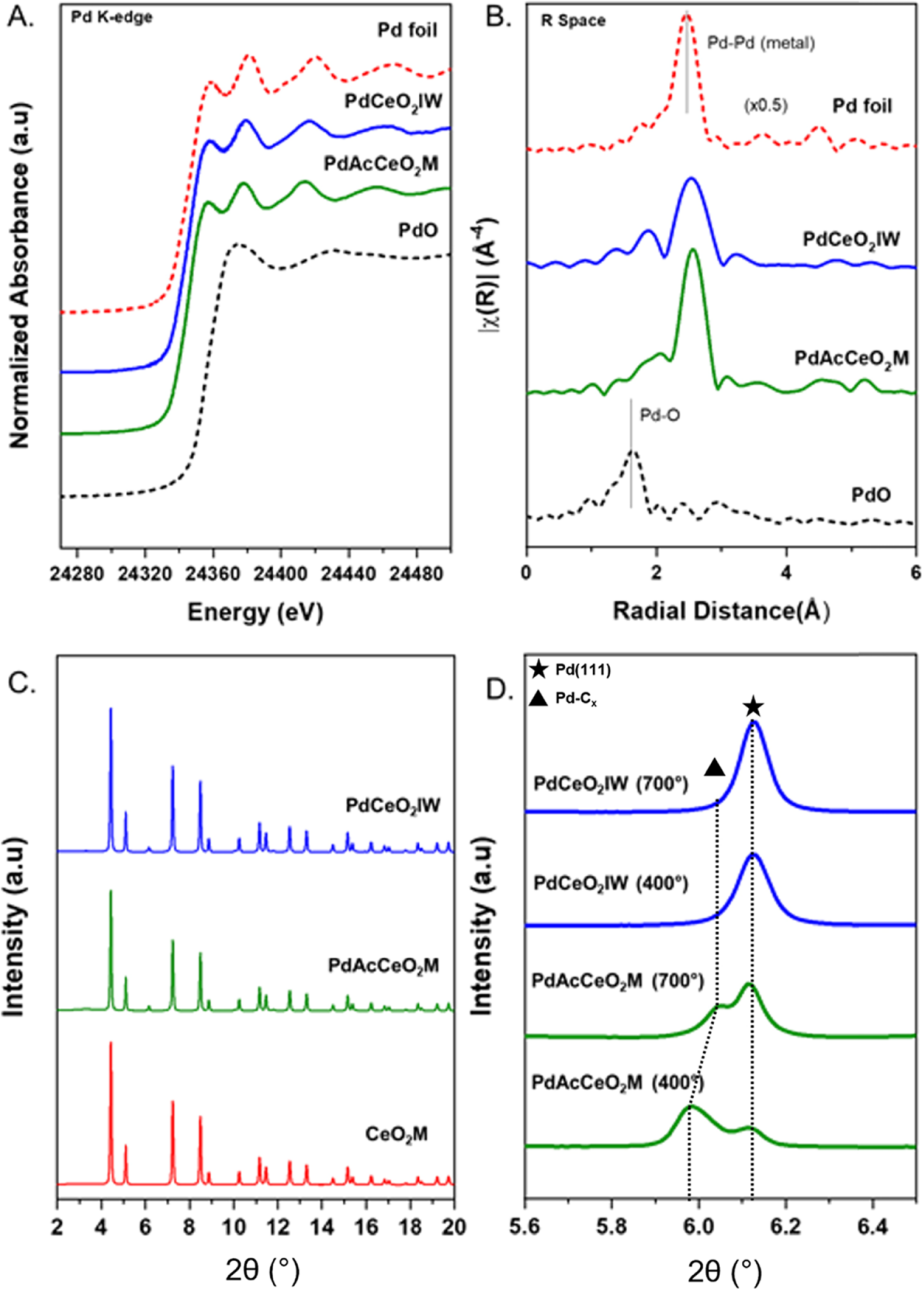
(A) XANES and (B) EXAFS spectra of the H_2_ reduced 4 wt % PdAcCeO_2_M and PdCeO_2_IW. (C) Diffraction patterns for the tested samples at RT after H_2_ pretreatment and (D) in situ diffraction patterns at 400 and 700 °C under a 25%CO_2_/25%CH_4_/50%He mixture.

In situ XAFS studies were performed at the Pd K-edge (24,350 eV) to obtain the chemical state and estimate the average particle sizes of the supported Pd species after hydrogen reduction (Figure 2A,B). The Pd K-edge XANES shows that the oxidation state of Pd is close to that of metallic Pd for both samples, according to the position of the white line and the absorption edge. The Fourier-transformed R-space EXAFS spectra (Figure 2B) together with the fitting results (Table S2) show the presence of only Pd-Pd first shell (bond distance ∼2.75 Å, coordination number (C.N.) ∼ 10.4) in the PdAcCeO_2_M with an average diameter of ∼5 nm for Pd nanoparticles estimated using a semisphere model.^35^ XRD patterns of the reduced Pd–CeO_2_ samples (Figure 2C) show multiple peaks associated with a CeO_2_ fluorite crystalline structure and a distinct Pd diffraction pattern corresponding to metallic Pd at 2θ = 6.1° (JCPDS 46-1043). The average ceria particle size is estimated to be 90–100 nm in diameter, consistent with the sizes obtained from TEM. Subsequent XRD analyses under reaction conditions at 400 and 700 °C are shown in Figure 2D, highlighting the splitting of the Pd peak whereby these additional Pd features were further examined using in situ XRD.

**Figure 3:**
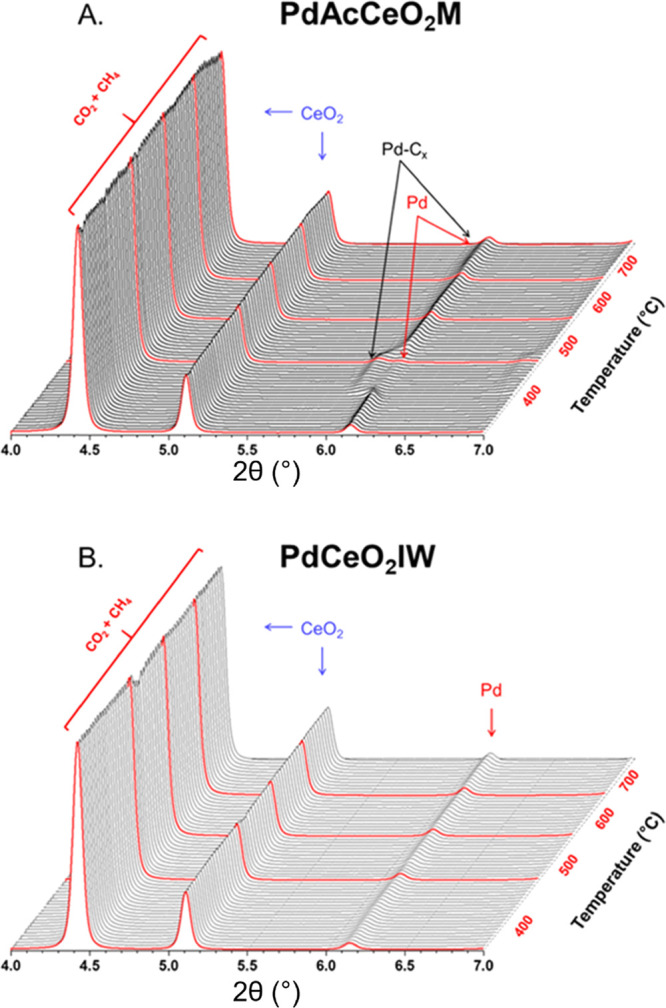
In situ XRD profiles of (A) PdAcCeO_2_M and (B) PdCeO_2_IW under DRM conditions. The catalyst was pretreated under H_2_ at 400 °C for 30 min before switching the gas to a 25%CO_2_/25%CH_4_/50%He mixture. Temperature was increased to 400 °C under steady-state conditions and further increased (100 °C every 30 min) until reaching 700 °C (red lines mark the first diffraction pattern collected after the temperature increase).

In situ XRD analysis was used to complement the reactivity findings for both samples. During reaction, the catalyst undergoes chemical and structural changes where in situ techniques are essential to understand the interplay between Pd and CeO_2_.^36,37^Figure 3A,B shows the continuous diffraction scans collected at each DRM step for both samples. For the in situ reaction, the catalyst was first pretreated from RT up to 400 °C under H_2_, then cooled down to RT where it was exposed to CO_2_ and CH_4_ and ramped up to temperature. The ceria fluorite-type crystal structure with (111) and (200) reflections at 4.4° and 5.2° 2θ does not show drastic changes as the temperature is increased, and Rietveld refinement of the CeO_2_ lattice parameter indicates exclusively thermal expansion effects (Figure S6).

After the pretreatment, under prolonged CO_2_ and CH_4_ exposure at 400 °C, on PdAcCeO_2_M the bulk Pd phase structure exhibits a unique splitting where a secondary peak at 2θ < 6.1° emerges and later decreases in intensity as the temperature is increased over 600 °C. Conversely, on the PdCeO_2_IW sample the pretreatment reduces PdO into Pd^0^, which remains stable under DRM conditions and undergoes no further structural changes (Figure 3B) except for an increase in the Pd (111) peak intensity as a result of sintering. The splitting of the Pd peak on the milled catalyst is a distinctive feature of this sample under reaction conditions. While this transition could theoretically resemble a PdO_2_ phase, which has been ascribed to unique low methane activation barriers,^38,39^ it is improbable that this oxide could form in the presence of such a mild oxidant as CO_2_ and after reduction under H_2_ at 400 °C. More likely, this feature emerges because of the incorporation of carbon onto Pd, and it is still present under reaction conditions up to 700 °C (Figure 2D).^40^ The presence of Pd–C_*x*_ formed under in situ DRM conditions has not been highlighted before on Pd–CeO_2_.^41^ However, the copresence of a carbide-like phase has been shown to improve the catalytic activity of other Pd-based catalysts, in particular for CO_2_ reforming of methane on a Pd/ZrO_2_ model catalyst^42^ and for ethylene hydrogenation on Pd/C.^43^ DRM catalysts with varied compositions, such as PdZrO_2_^42^ or NiFe-MgO,^44^ have reported an initial induction period to form a beneficial carbon overlayer that promotes CO_2_ reduction during DRM. Our findings are consistent with these evidences, where based on the time on stream study we observe an improvement in CO_2_ conversion as a function of time.

The nature of this Pd–C_*x*_ feature was carefully examined by in situ XRD under CH_4_ only, as it is known that the C–H bond cleavage is the limiting step for the DRM reaction. XRD profiles corresponding to the as-prepared PdAcCeO_2_M and PdCeO_2_IW under a reductive CH_4_ atmosphere (Figure S7) were taken ramping the temperature from 25 to 700 °C. The milled sample shows the presence of reduced Pd up to 300 °C, where a shift to lower 2θ is observed. This transition is also evident in the impregnated sample and corresponds to changes in the palladium crystal lattice caused by carbon incorporation from methane decomposition.^45^ The peak shift observed on the milled Pd catalyst closely resembles the position of the peak in Figure 3A between 400 and 500 °C. Carbon deposition under CH_4_ exposure gave rise also to additional phases, more evident for PdAcCeO_2_M (Figure S8), that form because methane can adsorb and dissociate as well as oxidize to carbon oxides on the surface.^46^ However, in the presence of CH_4_ only, the O fed by the reduction of bulk ceria^47,48^ is limited, thus leading to CH_*x*(a)_ and H_(a)_ species on the surface where C-buildup occurs, forming a stable carbide-like phase at higher temperature and giving rise to a unique arrangement for the milled sample. The ceria peaks (Figure S7) show a drastic shift to lower 2θ as the temperature increases and reaches 500 °C, corresponding to an increase in the CeO_2_ lattice parameter consistent with the partial reduction (Ce^4+^ to Ce^3+^) of ceria layers near the surface and indicating the removal of lattice oxygen for methane activation. Conversely, in situ XRD on both CeO_2_–M and PdOCeO_2_M (Figure S9) show no discernable change in the CeO_2_ lattice under a CH_4_ atmosphere at elevated temperature. Semiquantitative CH_4_-TPR measurements (Figure S10) confirm a higher CH_4_ uptake for PdAcCeO_2_M, which is consistent with carbon being deposited inside the Pd lattice observed by in situ XRD, giving rise to a unique structure as observed under dry reforming conditions.

### Chemical Speciation of Palladium and Ceria via AP-XPS

**Figure 4:**
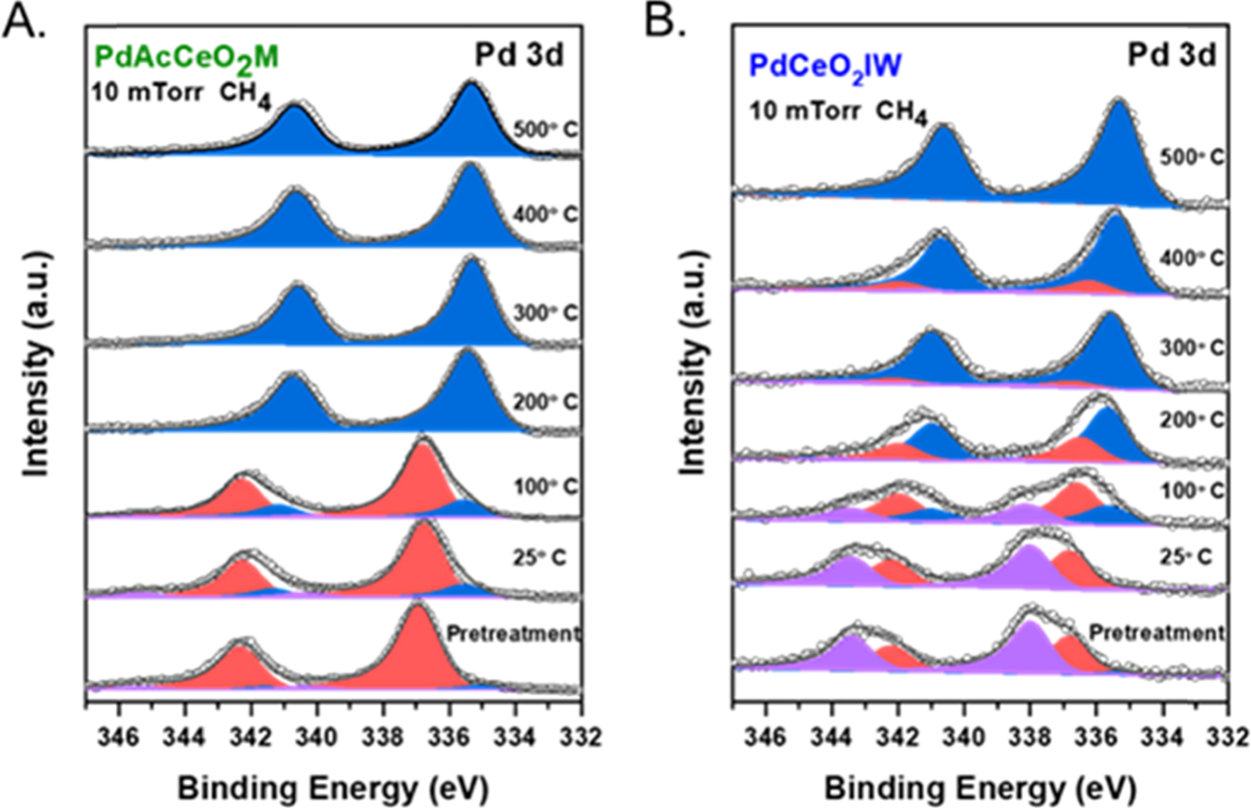
Pd 3d AP-XPS spectra of PdAcCeO_2_M (A) and PdCeO_2_IW (B) samples in a 10 mTorr CH_4_ atmosphere, from 25 to 500 °C, after O_2_ pretreatment where Pd^4+^, Pd^2+^, and Pd^0^ are colored in violet, red, and blue, respectively.

In situ AP-XPS was used to evaluate the surface-sensitive catalyst chemical state and surface species in the presence of methane. An O_2_ pretreatment was performed to follow the reduction of Pd species by CH_4_. After O_2_ exposure, the gradual surface changes upon exposure to 10 mTorr CH_4_ were monitored by AP-XPS on PdAcCeO_2_M and PdCeO_2_IW, collecting spectra in the Pd 3d and Ce 3d regions from 25 to 500 °C. The surface evolution of Pd species is displayed in Figure 4. On the milled sample, after pretreating the sample in O_2_, palladium is found mainly as Pd^2+^ at 25 °C. Between 100 and 200 °C, there is a clear reduction of the Pd oxide, with a peak shift of approximately 1.7 eV, consistent with the full reduction to metallic Pd associated with the activation of CH_4_. On the other hand, PdCeO_2_IW exhibits a more heterogeneous valence with a Pd^4+^/Pd^2+^ mixture after pretreatment in O_2_. After the removal of O_2_ and the introduction of 10 mTorr CH_4_ into the chamber, on PdCeO_2_IW reduction of oxidized Pd^4+/2+^ begins at 200 °C and is completed only at 500 °C, suggesting a slower H-spillover effect, in agreement with the smaller amount of activated methane observed by CH_4_-uptake experiments (Figure S10) and consistent with the structural differences observed by in situ XRD at elevated temperatures. At 500 °C (Figure S11), both materials show the formation of a carbide species at 284.3 eV, with a higher intensity for PdAcCeO_2_M consistent with the observations under XRD (Figure S7).

The Ce 3d spectra (Figure S12) were also collected upon CH_4_ exposure. On both samples, the reduction of Pd oxides is accompanied by the removal of lattice oxygen in ceria, evidenced by a Ce^4+^ reduction to Ce^3+^ which initiates between 100 and 300 °C, and progresses as the temperature reaches 500 °C. The reduction of PdO followed by the removal of lattice oxygen in the oxide support was also confirmed by in situ XRD-TPR (Figure S7). Unlike the chemical states of surface Pd, Ce 3d spectra display minor differences between the milled and the wet-synthesized samples, indicating that ceria reduction and consequent increase in the lattice parameter (Figure S9) are associated with oxygen transfer from CeO_2_ bulk. The improved reducibility in PdAcCeO_2_M is thus a result of methane being readily activated at the Pd surface and facile oxygen transfer from bulk CeO_2_, which takes place more readily on the milled catalyst.

The higher tendency of the PdAcCeO_2_M to activate methane observed by in situ XRD and AP-XPS is reflected by its catalytic activity under DRM conditions. The CO_2_ from the feed interacts with surface C decomposing into CO via the Boudouard reaction and yielding a higher CO_2_ conversion, as was observed in the reactivity results in Figure 1. The addition of CO_2_ into the feed gas causes the carbon to be further oxidized by O adatoms because of CO_2_ dissociation over the support or at the metal–support interface. We contrast this with a milled PdO onto CeO_2_ (PdOCeO_2_M), where its inability to activate CH_4_ is likely a consequence of poor metal–support interactions, as demonstrated by the absence of changes in the CeO_2_ lattice parameter in the CH_4_ atmosphere (Figure S9C), which makes it a poor catalyst for DRM.^19^

Under steady-state conditions (Figure S4), PdAcCeO_2_M suggests that its surface Pd–Ce interaction is not only able to resist deactivation but also likely activates side reactions for additional CO_2_ conversion and methane dissociation. HRTEM images (Figure S13) were obtained after 24 h DRM exposure, showing that the size of Pd nanoparticles on both PdAcCeO_2_M and PdCeO_2_IW remained stable, with a minor change from 3.5 to 3.3 nm and from 2.0 to 2.4 nm for the milled and impregnated catalysts, respectively (Table S3). Possibly, the presence of a mild oxidant such as CO_2_ helps prevent the sintering of metallic Pd, as Pd redispersion at elevated temperatures in an oxidative environment has been previously reported.^49^ Thermogravimetric analysis (TGA) and TPO analysis (Figure S14) on the postreaction samples show that PdAcCeO_2_M has considerably higher carbon buildup than PdCeO_2_IW, consistent with the formation of residual carbon species. Regardless of this carbon buildup on PdAcCeO_2_M, it still shows higher reactivity and H_2_/CO ratio than PdCeO_2_IW, suggesting that not all carbon deposition leads to catalyst deactivation.^50^ The absence of carbon in the HRTEM images can be ascribed to the localized environment probed by this technique and/or to the presence of carbon as subsurface, well-dispersed carbon species.

### DRM Mechanistic Insights via Isotopically Labeled In Situ DRIFTS

The metal–support interface at the nanoscale created during milling is likely key to promote unique methane activation pathways. To probe the nature of the active site and key Pd sites, post-DRM reaction and postmethane exposure CO titration were performed on both PdAcCeO_2_M and PdCeO_2_IW (Figure S15). Both PdAcCeO_2_M and PdCeO_2_IW show an initial formation of primarily linear Pd–CO and bridged Pd–CO; however, after exposure to either DRM conditions or elevated temperature CH_4_ exposure, the preferential formation of higher wavenumber linear Pd–CO at 2090 cm^–1^ is observed on PdAcCeO_2_M, while a greater relative ratio of lower wavenumber linear Pd–CO, at 2060 cm^–1^, is observed on PdCeO_2_IW. Because of the complexity of the nanocrystalline Pd surface, the precise speciation of linear Pd–CO at 2090 cm^–1^ is unclear, which can be due to defect sites, nanocrystalline (111)/(100) facets, stable Pd hydride species remaining after the H_2_ pretreatment, or CO linearly bound to C-modified Pd on the samples after CH_4_ and/or CO_2_ exposure.^51,52^ Furthermore, PdCeO_2_IW after the DRM reaction shows additional surface heterogeneity via distinct shouldering of the bridged Pd–CO at 1990 cm^–1^.

To further elucidate these aspects, isothermal isotopic exchange between CO_2_ and ^13^CO_2_ DRIFTS was used to probe the surface intermediates present under a steady CH_4_ flow on PdAcCeO_2_M, PdCeO_2_IW, and CeO_2_–M. At room temperature, carbon dioxide does not adsorb on supported palladium, and no dissociation was detected at the palladium surface.^53,54^ On the basis of steady-state IR spectra, 250 °C was chosen as the experimental temperature, as CO adsorption on Pd and formates in the form of bidentate oxo-species showed relatively high surface coverage and appropriate IR signals. Two major regions in the IR spectra were tracked: (1) the formation of CO species adsorbed on the metallic surface between 1900 and 2100 cm^–1^ and (2) adsorbed formates onto CeO_2_ between 1400 and 1600 cm^–1^ (Figure S16). The band at around 3575 cm^–1^ is assigned to associated hydroxyl groups.^55^ Assignments of the vibrational bands of different surface species are summarized in Table S4. A sharp peak located at 1595 cm^–1^ corresponding to a bidentate formate feature present on both PdAcCeO_2_M and CeO_2_–M confirms that this species is adsorbed on the CeO_2_ surface.^56−59^ The impregnated sample also exhibits this peak with the addition of many formates and carbonates as evidenced by several peaks in the 1300–1600 cm^–1^ region which reflect the C=O vibration modes of surface carbonates and other oxo-species.

**Figure 5:**
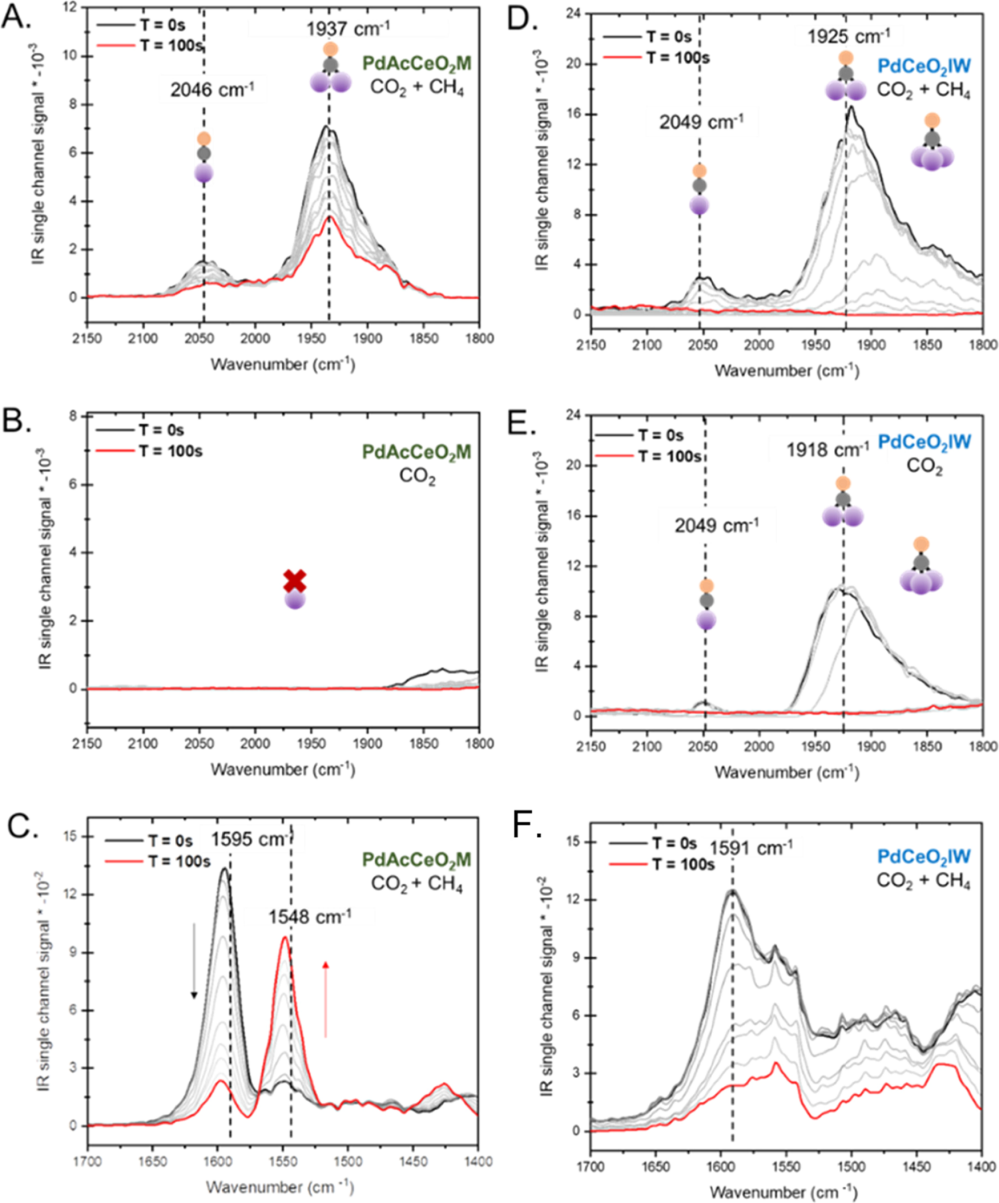
Isothermal before (black line), during (gray lines), and after (red line) ^12^CO_2_ to ^13^CO_2_ switching DRIFTS treatment of PdAcCeO_2_M and PdCeO_2_IW catalysts at 250 °C (CH_4_/^12^CO_2_ (^13^CO_2_)/He = 5/5/30 mL/min). PdAcCeO_2_M: Pd–CO region under (A) ^12^CO_2_/^13^CO_2_ and CH_4_ (B) CO_2_ (^12^CO_2_(^13^CO_2_)/He = 5/35 mL/min) and (C) formate region under ^12^CO_2_/^13^CO_2_ and CH_4_; PdCeO_2_IW: Pd–CO region under (D) ^12^CO_2_/^13^CO_2_ and CH_4_ (E) ^12^CO_2_/^13^CO_2_ and (F) formate region under ^12^CO_2_/^13^CO_2_ and CH_4_.

Figure 5A shows a close-up to the Pd–CO region for PdAcCeO_2_M where distinct Pd–CO adsorption peaks can be observed. The peak located at 2046 cm^–1^ is attributed to linear Pd^0^-CO, and the 1937 cm^–1^ feature is characteristic of bridged Pd–CO.^51,60^ Under isotopic switching from ^12^CO_2_ to ^13^CO_2_, the intensity of the Pd–CO decreases slightly, but this Pd–CO bond is still present after 100 s of switching. Another run was done by exposing the material to CO_2_ only. The removal of CH_4_ from the feed gas for PdAcCeO_2_M strongly suggests that CO is being formed because of the direct oxidation of CH_4_. In fact, without the presence of CH_4_ in the feed (Figure 5B) there is no CO formation, indicating that the Pd on PdAcCeO_2_M offers active sites for CH_4_ dissociation leading to a direct pathway for its oxidation. To directly evaluate the decomposition of CO_2_, the formate/carbonate region extending from 1700 to 1400 cm^–1^ was examined with isothermal isotopic switching DRIFTS at 250 °C in Figure 5C. The DRIFTS spectra features associated with bidentate formate adsorption were observed to shift to lower wavenumbers (from 1595 to 1548 cm^–1^) within approximately 2 min of the gas switch. The replacement of H^12^COO with H^13^COO (peaks at 1595 and 1548 cm^–1^, respectively) on the exchanged Pd sites in the ceria for PdAcCeO_2_M strongly suggests that this formate is actively participating in the reaction. Without the presence of CH_4_ (Figure S17), there is no scrambling of the H^13^COO peak, indicating that this bidentate formate is associated with the reaction mechanism and interacting with adsorbed Pd–CH_*x*,_ which is necessary for the proposed pathway for this material to form Pd–CO.

Afterward, surface migration of formate toward the Pd–CeO_2_ interface likely takes place, decomposing via Pd bound carbonyl intermediates. Note that without CH_4_ in the feed, there is no CO adsorption or a route to decompose the surface formate feature on the CeO_2_. The proposed mechanism for the reaction in the PdAcCeO_2_M sample is then as follows:
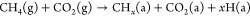
1
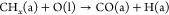
2
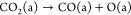
3
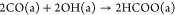
4
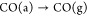
5
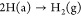
6
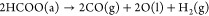
7
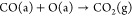
8
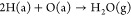
9

In contrast, for the Pd–CO region, PdCeO_2_IW shows both linear and bridged Pd–CO, and the band extends to CO being bound on hollow Pd sites (Figure 5D).^51,60^ Under isotopic switching from ^12^CO_2_ to ^13^CO_2,_ the intensity of the Pd–CO drops to zero abruptly, indicating that CO_2_ is likely responsible for Pd–CO bond formation. Under CO_2_ only conditions (Figure 5E), there is no change in the Pd–CO adsorption features indicating that this catalyst prefers to use Pd sites for CO_2_ reduction. This CO_2_ reduction pathway with the H_2_ being formed from CH_4_ decomposition is likely responsible for the formation of stable polydentate carbonates over the PdCeO_2_IW catalyst (Figure 5F), which is undesirable for low-temperature activity and CO_2_ dissociation where there is no scrambling of the carbonate peak. By isotopic DRIFTS measurements, it is then possible to identify specific active sites and obtain evidence of an active mechanism for the CH_4_ conversion pathway in the PdAcCeO_2_M under the presence of CO_2_, which is entirely different from the one taking place on the surface of PdCeO_2_IW.

**Figure 6:**
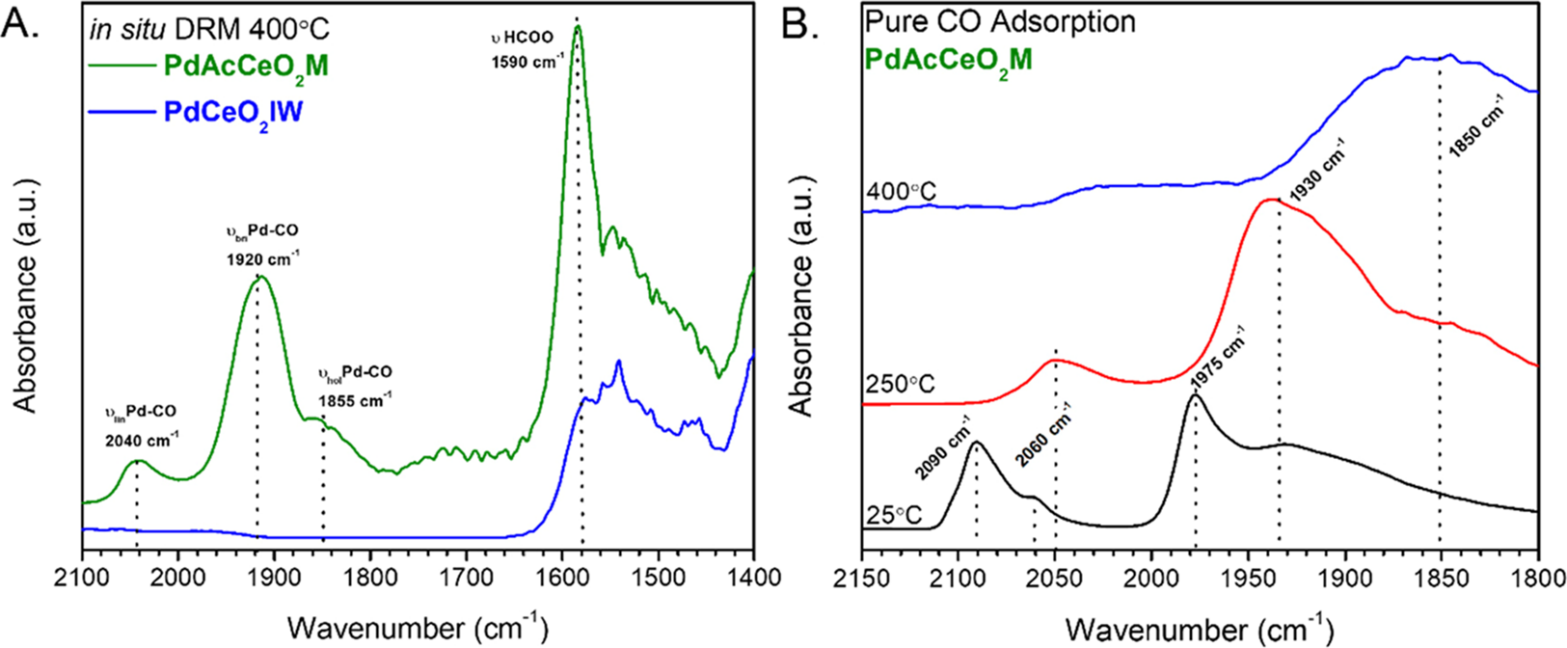
In situ Pd–CO interactions under (A) in situ DRM conditions (CH_4_/CO_2_/He = 5/5/30 mL/min) at 400 °C on PdAcCeO_2_M and PdCeO_2_IW and (B) PdAcCeO_2_M after an initial 400 °C H_2_ pretreatment at varied CO adsorption temperatures, 10%CO/He dosing and subsequent He purge, where the spectra were collected at the listed temperature.

The role of Pd in PdAcCeO_2_M is therefore to dissociate CH_4,_ generating oxygen vacancies while the CO_2_ is adsorbed on basic adsorption sites on CeO_2_. This is supported by in situ DRM DRIFTS performed at 400 °C on both catalysts, which clearly show that a similar trend is observed on PdAcCeO_2_M at elevated temperatures; specifically, the formation of Pd–CO intermediates and the propagation of HCOO, shown in Figure 6A. However, PdCeO_2_IW shows a notable absence of HCOO and no appreciable Pd–CO formation. The stoichiometric adsorption of CO on PdAcCeO_2_M at various temperatures, in Figure 6B, shows that in the absence of DRM conditions linear Pd–CO is not a prominent surface species at either 250 or 400 °C, highlighting the critical role of DRM conditions in the formation of the Pd–CO intermediates. The relative binding strength of stoichiometrically adsorbed Pd–CO scales according to: ν_hol_Pd–CO > ν_bri_Pd–CO > ν_lin_Pd–CO; with linear Pd–CO (2030 cm^–1^ ≥ ν ≥ 2090 cm^–1^) readily desorbing at higher temperatures while the hollow site Pd–CO centered at ∼1850 cm^–1^ persists, even at 400 °C. Room-temperature CO adsorption on both PdAcCeO_2_M and PdCeO_2_IW (Figure S18) after a H_2_ pretreatment shows the speciation of the bridge Pd–CO site on PdCeO_2_IW, which can attributed to the selective adsorption of CO on either Pd(100) at ∼1980 cm^–1^ and the adsorption on Pd(111) at ∼1940 cm^–1^.^52,61^ This indicates that despite taking into account the effect of temperature,^62^ CO chemisorption is not a representative gauge of transient CO produced from selective CH_4_ oxidation in the milled catalyst or CO being produced from the CO_2_ reduction route in the impregnated sample.

These findings corroborate that the DRM mechanism proceeds via CH_4_ adsorption over the active Pd sites to form carbon and H_2_. The resulting carbon is further oxidized by O adatoms as a result of CO_2_ dissociation either over the support or at the metal–support interface.^63,64^ The reaction of CH_*x*_ fragments with CO_2_ and surface adsorbed oxygen (O_ads_) is very efficient on the milled sample, limiting the decomposition of CH_*x*_ to surface carbon. A similar mechanism for DRM over Ni-based catalysts has been previously proposed where the formate species form via O* addition to adsorbed CH_x_* species, where the decomposition of CH_*x*_ to surface carbon was found to be detrimental to reactivity.^65,66^ These results highlight that not only is the nature of the support essential for promoting metal–support interplay,^67^ but that the nanoscale interactions between the metal and substrate can influence and, in turn, enhance both CH_4_ conversion pathways and simultaneous CO_2_ activation.

### DFT Theoretical Modeling of CO on Pd Surfaces

To gain better understanding of the in situ DRIFTS results (Figure S15) and therefore identify the DRM-driven surface structure of Pd in PdAcCeO_2_M and PdCeO_2_IW samples, DFT calculations were performed to study the CO adsorption on bare Pd(111) and Pd(111) covered by chemisorbed hydrogen, carbon, and oxygen, which could be produced during DRM. This includes hydrogen with a coverage of one monolayer (ML) [Pd(111)_H-1ML_ in our notation] associated with favorable methane dissociation, 1 ML of oxygen with a coverage of 1 ML [Pd(111)_O-1ML_] associated with facile CO_2_ dissociation, and carbon with a coverage of 0.25, 0.5, and 1 ML associated with preferential methane dissociation over carbon oxidation. In addition, the bulk hydride [PdH(111)] and oxidized [PdO(111)] surfaces were also considered as extreme cases (Figure S19). A detailed discussion of the DFT modeled sites, including Pd(111)_H-1ML_, Pd(111)_O-1ML_, and Pd(111)_C-(0.25–1.0)ML_, is presented in the Supplementary Information along with the top and side views of CO adsorption (Figure S20).

The DFT-calculated adsorption energies of *CO at the possible sites on various Pd(111)-based surfaces are listed in Table 1, along with the corresponding C–O stretching frequencies. On Pd(111), the most stable binding site for CO is the Pd fcc hollow (Pd_3_-fcc in our notation) site (B.E. = −1.95 eV) with a C–O bond stretching frequency of 1808 cm^–1^, which is followed by that on the Pd bridge site (Pd_2_-bridge, B.E. = −1.79 eV, ν = 1881 cm^–1^) and Pd top site (Pd_1_-top, B.E. = −1.36 eV, ν = 2049 cm^–1^) via a decreasing sequence in binding strength and an increasing sequence in frequency, which agree reasonably well with the experimental values.^52,61^

**Table 1:** DFT-Calculated CO Binding Energy and Corresponding C–O Stretching Frequency on Pd(111), Pd(111)_H-1ML_, PdH(111), Pd(111)_O-1ML_, PdO(111), Pd(111)_C-0.25ML_, Pd(111)_C-0.5ML_, and Pd(111)_C-1ML_ Surfaces

surface	binding site	B.E. (CO) (eV)	ν(C–O) (cm^–1^)
Pd(111)	Pd_3_-fcc	–1.95	1808
	Pd_2_-bridge	–1.79	1881
	Pd_1_-top	–1.36	2049
Pd(111)_H-1ML_	Pd_3_-fcc	–0.25	1862
	Pd_2_-bridge	–0.28	1966
	Pd_1_-top	–0.22	2066
PdH(111)	Pd_3_-fcc	–0.58	1867
	Pd_2_-bridge	–0.64	1930
	Pd_1_-top	–0.46	2068
Pd(111)_O-1ML_	O_2_-bridge (carbonate)	–3.29	1631
PdO(111)	no adsorption		
Pd(111)_C-0.25ML_	Pd_3_-hcp	–0.93	1860
	Pd_2_-bridge	–0.90	1957
	Pd_1_-top	–1.09	2057
	C_1_-top	–1.29	2105
Pd(111)_C-0.5ML_	C_1_-top	–1.86	2105
Pd(111)_C-1.0ML_	C_1_-top	–2.63	2119

By comparing the C–O stretching frequency from DRIFTS-measured CO titration under post-DRM conditions (Figure S15) and DFT calculations on various Pd(111)-based surfaces (Table 1), a better understanding of the surface phase can be achieved. The peak around 1920 cm^–1^ is likely associated with the CO adsorption on partially reduced Pd^δ−^ sites on PdH(111) surface, specifically the most stable Pd_2_-bridge (ν = 1930 cm^–1^). By comparison the contribution from Pd_H-1ML_(111) is likely less due to its weaker binding of CO (<−0.3 eV). This is consistent with the experimental observations on the pure H_2_-pretreated PdAcCeO_2_M, which readily desorbs CO after extended purging post CO adsorption. The peak centered at 1975 cm^–1^ can be attributed to CO at the Pd sites on Pd_C_(111), which are partially oxidized by carbon, specifically the Pd_2_-bridge site on Pd_C-0.25ML_(111) (ν = 1957 cm^–1^). The peak at 2060 cm^–1^ may represent the combination of CO adsorbed at the top site of metallic Pd^0^ [Pd_1_-top on Pd(111): ν = 2068 cm^–1^] and partially oxidized Pd^δ+^ by carbon [the most stable Pd site, Pd_1_-top, on Pd_C-0.25ML_(111): ν = 2057 cm^–1^]. Finally, the highest frequency around ∼2100 cm^–1^, present as a slight shouldering of the 2090 cm^–1^ peak on only PdAcCeO_2_M, is possibly a fingerprint for the C_1_-top adsorption and formation of *CCO on Pd_C_(111) surfaces [Pd_C-0.25ML_(111) and Pd_C-0.5ML_(111): ν = 2105 cm^–1^], which is more favorable to stabilize CO than that on the Pd sites. The PdO(111) and Pd_O-1ML_(111) surfaces are ruled out, as on exposure to CO, the oxidized Pd(111) by oxygen can be difficult to form due to the facile CO oxidation to CO_2_ as indicated above and PdO(111) does not adsorb CO at all.

Overall, the DFT predicts the coexistence of multiple surface Pd species in both PdAcCeO_2_M and PdCeO_2_IW under post-DRM conditions, including metallic Pd^0^, partially reduced Pd^δ−^ in hydride, and partially oxidized Pd^δ+^ in carbide. Both Pd^0^ and Pd^δ−^ species can be well observed in both PdAcCeO_2_M and PdCeO_2_IW, where a similar profile is shown for the C–O stretching on CO titration (Figure S15). A significant difference is featured by the relative intensity on the 2090 cm^–1^ to 2060 cm^–1^ linear Pd–CO peak for PdAcCeO_2_M rather than PdCeO_2_IW. This species can thus be tentatively ascribed to the partially oxidized Pd^δ+^ in carbide predicted by DFT calculations. According to in situ XRD, TGA, and TPO experiments under reaction conditions PdAcCeO_2_M takes up carbon more significantly than PdCeO_2_IW, and the synergy between the distinct Pd^δ+^ and carbon sites on PdAcCeO_2_M may contribute to the enhanced DRM compared to PdCeO_2_IW as observed experimentally (Figure 1).

## Conclusions

By coupling in situ characterization with isotopic DRIFTS measurements, we have been able to identify the origin of the unique activity and stability of a mechanochemically synthesized PdAcCeO_2_M catalyst, which showed a higher reaction yield for H_2_ and CO compared to an impregnated PdCeO_2_IW sample. In situ measurements, carried out up to almost the temperature limit of in situ techniques (700 °C), revealed the carbon-modified Pd^0^ and Ce^4+^ as the active phase under these reaction conditions. The nanoscale Pd–Ce interplay also plays a key role in the resistance to deactivation of PdAcCeO_2_M. Under methane gas only, the importance of the Pd–Ce surface arrangement for the C–H bond activation becomes even more evident where metal–support effects greatly influence CH_4_ uptake. PdAcCeO_2_M undergoes substantial transformations even at room temperature, leading to an outstanding methane activation performance. In situ DRIFTS with isotopic exchange was able to clearly distinguish different DRM reaction pathways on the milled and impregnated catalysts. For the milled catalyst, Pd–CO was observed as an important surface species that arises directly from C–H oxidation. This Pd–CO species remains stable under reaction conditions and, in turn, lends to HCOO species that are a result of low-temperature CO_2_ reduction distinctly attributed to the chemistry of the milled sample, yielding enhanced CO + H_2_ production. The identity of the surface was also corroborated via DFT, which shows that the active phase is a partially oxidized Pd^δ+^ modified by adjacent carbon to result in unique Pd ensembles, where PdAcCeO_2_M has a stronger affinity toward carbon formation relative to PdCeO_2_IW under reaction conditions, suggesting that the carbon-modified Pd is a beneficial active site for DRM.

## Supporting Information Available

Details of Weisz–Prater and Anderson criteria analysis and DFT results, measured H_2_, CO, and H_2_O reaction rates during the DRM reaction (Figure S1), reverse water gas shift activity (Figure S2), 24 and 40 h stability experiments under DRM conditions (Figures S3 and S4), summary of state-of-the-art DRM catalysts (Table S1), HRTEM images of H_2_ reduced PdAcCeO_2_M and PdCeO_2_IW at 400 °C (Figure S5), Pd K-edge EXAFS fitting results (Table S2), in situ XRD Rietveld refinement (Figure S6), time-resolved and last cycle in situ XRD CH_4_-TPR profiles for PdAcCeO_2_M and PdCeO_2_IW (Figures S7, S8), in situ XRD CH_4_-TPR profiles for PdOCeO_2_M and CeO_2_–M and Rietveld refinement (Figure S9), semiquantitative CH_4_-TPR uptake measurements (Figure S10), in situ CH_4_-TPR XPS C 1s and Ce 3d spectra (Figures S11, S12), HRTEM of spent PdAcCeO_2_M and PdCeO_2_IW after DRM exposure at 700 °C (Figure S13), TGA/TPO of spent DRM catalysts after 24 h (Figure S14), particle size after different pretreatments (Table S3), CO-chemisorption DRIFTS spectra after exposure to different reaction atmospheres (Figure S15), in situ DRIFTS spectra under DRM exposure (Figure S16), assignment of IR bands (Table S4), time-resolved in situ FTIR difference spectra (Figure S17), DRIFTS spectra of CO adsorption on PdCeO_2_ materials at RT (Figure S18), top view of DFT-optimized surfaces (Figure S19), and top and side views of DFT-optimized *CO adsorption on different Pd sites (Figure S20) (PDF)
