# Supplementary material for: Identification of Highly Selective Surface Pathways for Methane Dry Reforming Using Mechanochemical Synthesis of Pd–CeO2
Source: ACS Catal. 2022 Oct 7;12(20):12809–22. doi: 10.1021/acscatal.2c01120 (PMC9595205; doi:10.1021/acscatal.2c01120)
Supplement: Supplementary file 1 — cs2c01120_si_001.pdf [file cs2c01120_si_001.pdf]

Identification of Highly Selective Surface Pathways for Methane Dry Reforming using Mechano-Chemical Synthesis of Pd-CeO_2

Supplementary Information

Juan D. Jiménez[1†], Luis E. Betancourt[1†], Maila Danielis[2], Hong Zhang[3], Feng Zhang[3], Ivan Orozco[3], Wenqian Xu[4], Jordi Llorca[5], Ping Liu[1,3], Alessandro Trovarelli[2], José A. Rodríguez[1,3], Sara Colussi[2], and Sanjaya D. Senanayake*[1]*

[1] Chemistry Division, Brookhaven National Laboratory, Upton, NY, 11793 USA

[2] Polytechnic Department and INSTM, University of Udine, Via del Cotonificio 108, 33100 Udine, Italy

[3] Department of Chemistry, State University of New York Stony Brook, Stony Brook, NY, 11794 USA

[4] X-ray Science Division, Advanced Photon Source, Argonne National Laboratory, Lemont, IL, 60439 USA

[5] Institute of Energy Technologies and Department of Chemical Engineering, Universitat Politécnica de Catalunya, EEBE, Eduard Maristany 10-14, 08018 Barcelona, Spain

† Authors Contributed Equally

* Corresponding Authors: ssenanay@bnl.gov, sara.colussi@uniud.it.

Weisz-Prater and Anderson Criteria Analysis

The internal mass transfer limitation of the system was explored via a Weisz-Prater Criterion analysis where the LHS of the equation should be ≪1.0 to rule out internal mass transfer limitations:

$$\frac{r_A' \rho_c R_p^2}{D_e C_{AS}} \ll 1.0$$

Where r_A' is the measured reaction rate, ρ_c is the pellet density, R_p is the pellet radius, D_e is the effective diffusivity, and C_{AS} is the surface concentration. D_e is given by equation 2

$$D_e = \frac{D_{CH4-N2} \varphi_P \sigma}{\tau}$$

Where φ_p is the pellet porosity, σ is the constriction factor, and τ is the tortuosity and $D_{CH4\text{-}N2}$ is the diffusion coefficient. A general approximation of φ_p=0.4, σ=0.8, and τ=3 was used for the catalyst. The diffusion coefficient for CH_4 and N_2 is given by:

$$D_{CH4-H2} = \frac{0.00266 T^{3/2}}{P M_{CH4-N2}^{1/2} \sigma_{CH4-N2}^2 \Omega_D}$$

where T is the temperature (K), P is the pressure (bar), $M_{CH4\text{-}N2}=2*[(1/M_{CH4})+(1/M_{N2})]^{-1}$, M_{CH4}=molecular weight of CH_4, M_{N2} = molecular weight of N_2, σ_{CH4-N2} = characteristic length (Å) and Ω_D is the diffusion collision integral (dimensionless). $\Omega_D$is given by Neufeld's approximated diffusion collision integral.[1]

$$\Omega_D = \frac{A}{(T^*)^B} + \frac{C}{\exp(DT^*)} + \frac{E}{\exp(FT^*)} + \frac{G}{\exp(HT^*)}$$

Where T* is $kT/\varepsilon_{CH4\text{-}N2}$, where $\varepsilon_{CH4\text{-}N2}$ is the characteristic energy, J, and the rest are known constants. Ω_D was found to be 0.749. $D_{CH4\text{-}N2}$ was found to be 1.61×10^{-4} m^2/s, De was 1.71×10^{-5} m^2/s, ρ_c was 7.6×10^3 kg/m^3, R_p was ~1.07×10^{-4} m, C_{CH4} was 13.26 mol/m^3, and r_{CH4}' at 700 °C was 0.234 mol/kg/s for $PdAcCeO_2M$ and 0.131 mol/kg/s for $PdCeO_2IW$. The LHS of the Weisz Prater

Criterion was found to be 0.09 and 0.05 for $PdAcCeO_2M$ and $PdCeO_2IW$, respectively, which satisfies the Weisz-Prater Criterion in the high flow, high conversion regime. Furthermore, using the CO_2-N_2 binary system and the relevant equations shown above with a rate of CO_2 conversion of 0.310 mol/kg/s and 0.231 mol/kg/s at 700 °C for $PdAcCeO_2M$ and $PdCeO_2IW$, respectively, the LHS of the Weisz Prater criterion are 0.16 and 0.11 for $PdAcCeO_2M$ and $PdCeO_2IW$, respectively. Therefore, the system is not mass transfer limited in either CH_4 or CO_2.

To exclude the presence of internal heat transfer limitations the Anderson criterion was used[2]

$$\frac{|\Delta H_{Rx}| r_{CH4} \rho_c R^2}{\lambda^e T_s} < 0.75 \frac{R_g T_s}{E}$$

where ΔH_{Rx} is the reaction enthalpy (260 kJ/mol), r_{CH4}' is the observed reaction rate, ρ_c is the catalyst density, R is the pellet size radius, T_s is the reaction temperature (973 K) and R_g is the gas constant (8.314 J/(mol·K)). λ^e and E represent the effective thermal conductivity and the experimental activation energy, respectively, for which approximate values were used (11.71 W/(K·m) and 70 kJ/mol, respectively).[3] Using these parameters, we obtain values of $5.87 \cdot 10^{-4}$ and $4.4 \cdot 10^{-4}$ for the left term (for $PdAcCeO_2M$ and $PdCeO_2IW$, respectively) and a value of 0.09 on the right term. The criterion is thus satisfied. In addition, it is worth noting that DRM activity tests were performed on 10 mg of powder catalyst diluted with pre-calcined SiO_2 in a 1:2 ratio, as to further reduce the risk of mass and heat transfer limitations.

DFT results

Each surface is relaxed and minimized for surface species prior adsorption of CO, it is found H, O and C all prefer the fcc site on Pd(111), only except C prefers the hcp site at low coverage, determined by adsorption energy. That is because the fcc site has more fluxionality upon adsorption of C. The horizontal Pd-Pd bond length is 2.794 Å on clean Pd(111), these bonds nearby C adsorption site at low coverage can be slightly extended when C falls in the hollow because the nearby hollows are empty. In case of carbon adsorbed at fcc site, they extended to 2.857 Å (average), and 2.839 Å in the case of hcp site, that means a more Pd-Pd bond tension when adsorbing CO at fcc site which destabilized the surface a bit and further reduced overall adsorption energy. At higher coverage, the Pd-Pd horizontal bond length restraints 2.794 Å nearby hollow sites are also occupied. But regardless of which hollow carbon prefers to stay, the interaction of CO and the surface is not affected because they take place on atop sites.

When the surface is fully covered by hydrogen [$Pd(111)_{H\text{-}1ML}$, B.E.: -0.25 ~ -0.22 eV, ν: 1862 ~ 2066 cm^{-1}] or the hydride is formed [PdH(111)), B.E.: -0.64 ~ -0.46 V, ν: 1867 ~ 2068 cm^{-1}], the CO binding is greatly weakened together with the increase in vibrational frequency and the site-dependency of CO binding energy is decreased (Table 1). Note that, since the surface is fully covered by hydrogen, to adsorb CO at Pd-hollow site requires the adsorbed hydrogen (*H) to be pushed away from the original hollow site (Figure S20), which makes it slightly less active than the Pd-bridge site (Table 1) with less shift of *H. Given that, the corresponding C-O stretching frequency is still higher than that at the Pd-hollow site.

On the oxygen-covered surface [$Pd_{O\text{-}1ML}(111)$], the CO adsorption leads to the oxidation to carbonate via C-O bond association (*CO_3, Figure S20). As a result, the corresponding binding is very strong (B.E. = -3.29 eV) with low C-O stretching of 1631 cm^{-1}. By comparison, when the

surface is fully oxidized [PdO(111)] it is less active with no CO adsorption due to the higher stability of surface oxygen. Note that the partially covered Pd(111) surface was not considered. Wherein the adsorbed *CO at Pd sites is not likely to survive but favoring the oxidation by neighboring *O to form CO_2 with an energy release of 0.6 eV.

In the case that the carbon build-up occurs on the surface during the DRM process [$Pd_C(111)$], at *C coverage of 0.25ML CO adsorbed on the Pd-top (B.E. = -1.09 eV, ν = 2057 cm^{-1}) next to the adsorbed *C is slightly more stable than that on Pd-hollow (B.E. = -0.93 eV, ν = 1860 cm^{-1}) and Pd-bridge (B.E. = -0.90 eV, ν = 1975 cm^{-1}). Like the case of $Pd_H(111)$, the CO-Pd interaction on $Pd_{C\text{-}0.25ML}(111)$ is weaker than that on Pd(111), while the C-O stretching frequencies only vary slightly. Besides, the minimal shift of *C away from the original hollow site on the adsorption of CO at the neighboring Pd sites can result in stronger CO binding at the lower symmetric sites. In contrast, the carbon itself can act as an active site for CO, and the C-top (B.E. = -1.29 eV, ν = 2105 cm^{-1}) is the most stable CO adsorption site on $Pd_{C\text{-}0.25ML}(111)$ via the formation of ketone-like *CCO species. With the coverage greater than 0.5 ML, the preference of *CCO over *CO is greatly increased, where spontaneous shift of *CO at Pd sites to the neighboring carbon site is observed during the geometry relaxation. This is associated with the decrease in number of available Pd sites and an increase in CO binding (B.E. = -1.86 eV, ν = 2105 cm^{-1} for $Pd_{C\text{-}0.5ML}$, B.E. = -2.63 eV, ν = 2119 cm^{-1} for $Pd_{C\text{-}1ML}$) are less accessible and the strengthening in C-CO interaction with increasing *C coverage. However, the promoted CO binding at high *C coverage in this case is likely associated with the increased distortion of surface *C species on interaction with CO. Thus, the corresponding C-O stretching frequency does not vary significantly once *CCO species are formed.

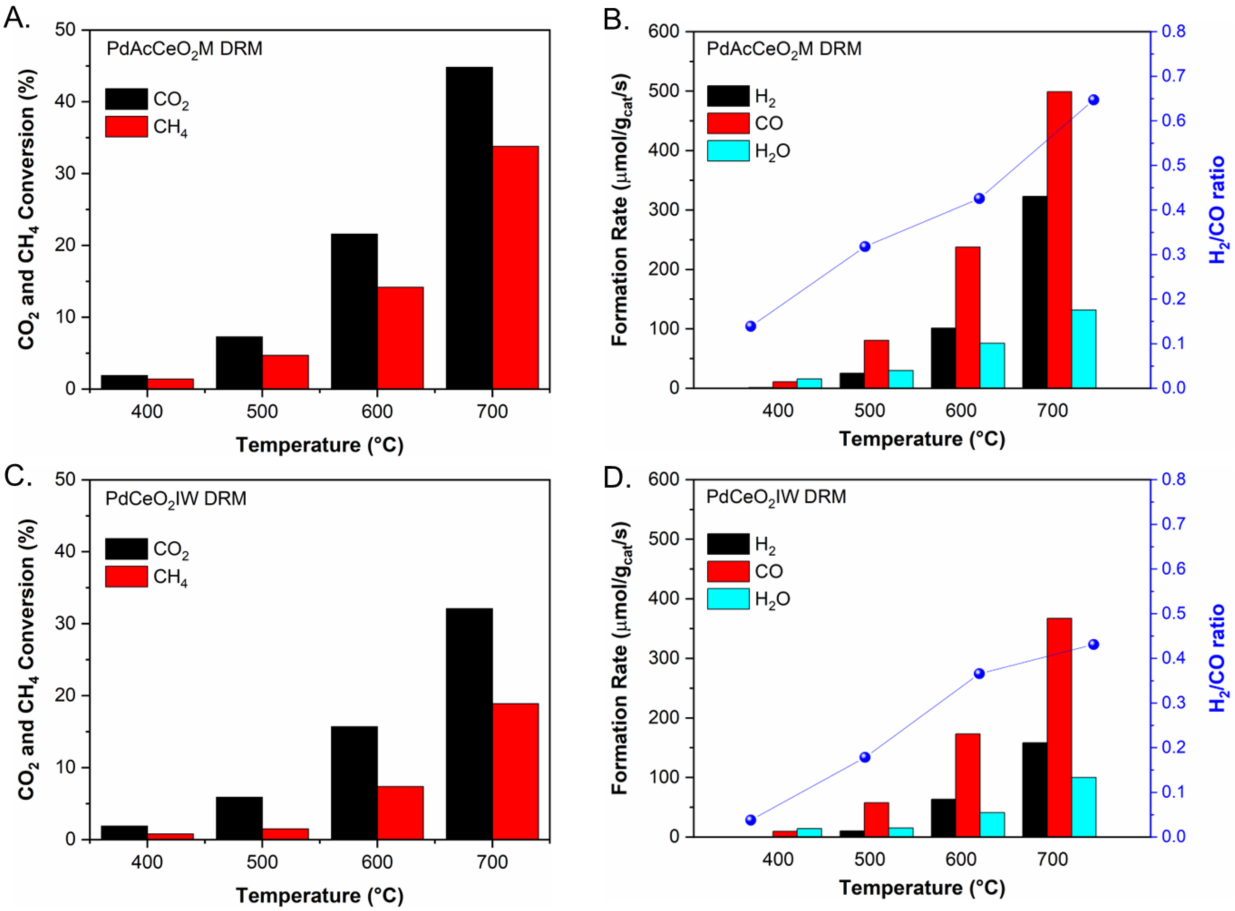

Figure S1. Comparison of the measured H_2, CO and H_2O reaction rates during DRM reaction over PdAcCeO_2M (A,B) and PdCeO_2IW (C,D). Reaction conditions: 10 mL/min CO_2 + 10 mL/min CH_4 + 10 mL/min N_2 with 10.0 mg of the catalyst; weight hourly space velocity (WHSV): 180,000 mL/(g_{cat}·h).

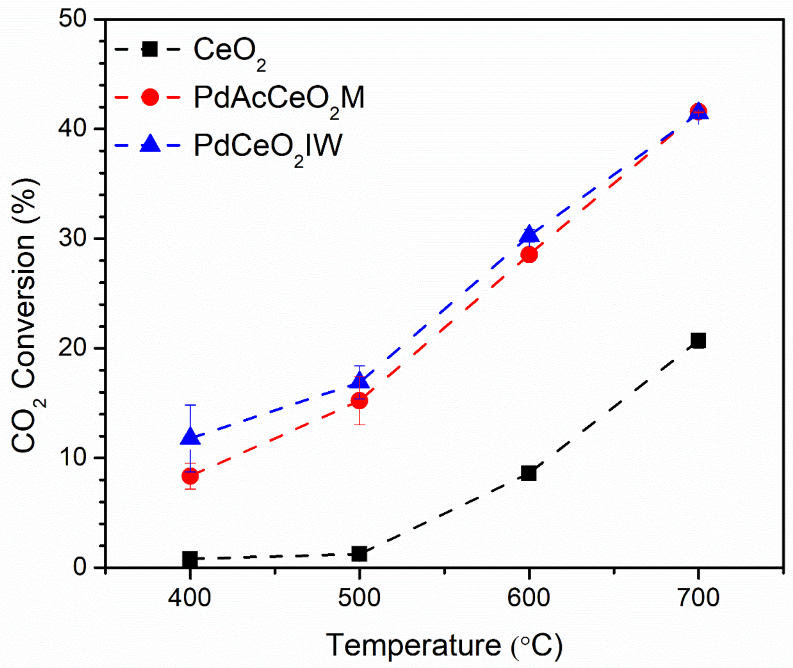

Figure S2. Reverse water gas shift reaction over $PdAcCeO_2M$, $PdCeO_2IW$ and blank CeO_2. CO selectivity >95% over all catalysts between 400-700 °C. Conditions: 1:1:1 ratio of CO_2:H_2:N_2 at a WHSV of 180,000 mL/(g_{cat}·h) with 10 mg of catalysts loaded, 400 °C H_2 pretreatment for 1 h on all catalysts, online analysis via GC.

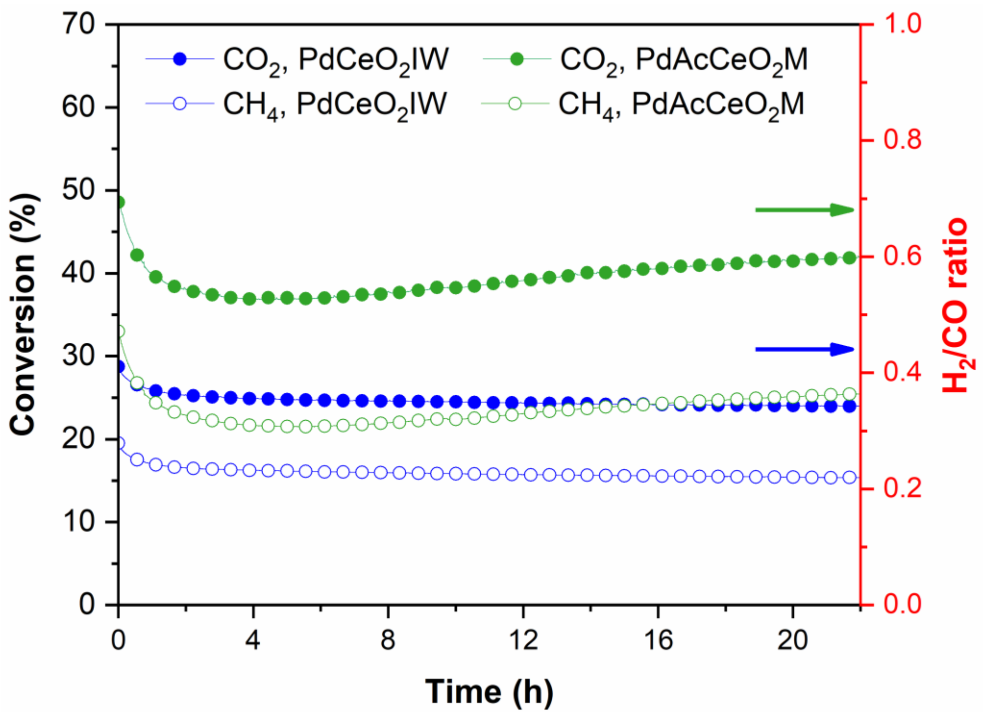

Figure S3. Catalytic stability test in DRM conditions for 24 h at 700 °C on the $PdAcCeO_2M$ (green) and $PdCeO_2IW$ (blue) samples; solid dots: CO_2 conversion, open dots: CH_4 conversion. H_2/CO ratio is marked by a green arrow ($PdAcCeO_2M$) and blue arrow ($PdCeO_2IW$); the H_2/CO ratio remained constant throughout the experiment.

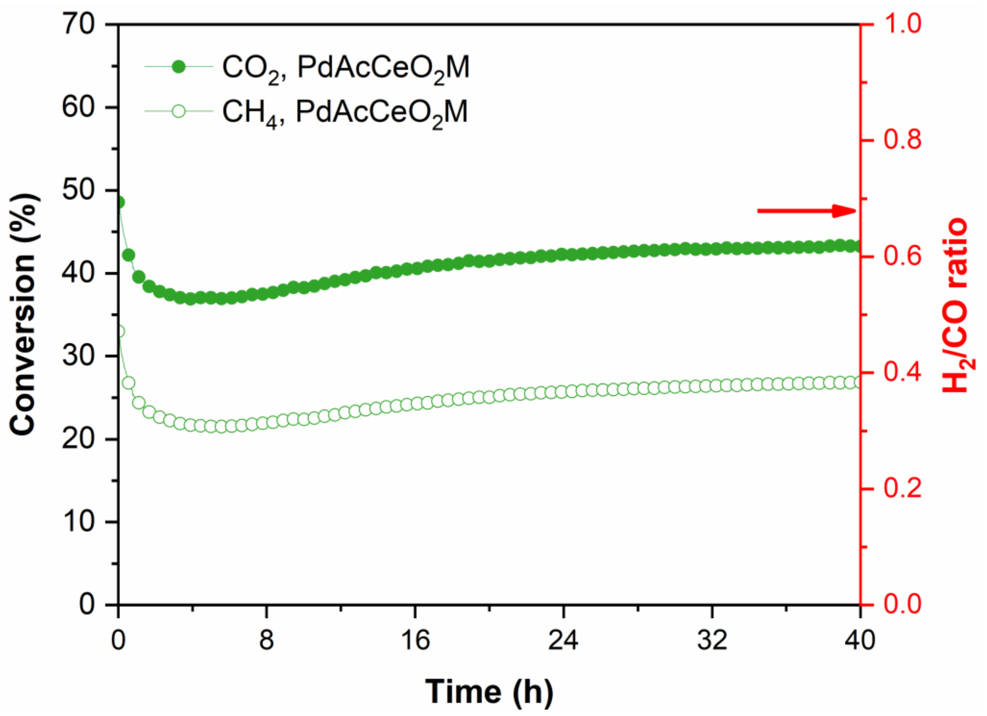

Figure S4. Catalytic stability test in DRM conditions for 40 h at 700 °C on $PdAcCeO_2M$. Solid dots: CO_2 conversion; open dots: CH_4 conversion; red arrow: H_2/CO ratio (constant throughout experiment).

Table S1. Comparison of catalytic activity for selected catalysts for DRM.

Catalysts	Temperature °C	WHSV $mL \cdot g_{cat}^{-1} \cdot h^{-1}$	Metal Loading	Rate of CH_4 Reaction $mol_{CH4} \cdot g_{cat}^{-1} \cdot s^{-1}$	Ref
$PdAcCeO_2M$	700	180000	4wt%Pd	2.3×10^{-4}	This Work
$PdCeO_2IW$	700	180000	4wt%Pd	1.3×10^{-4}	
$Ni/Mg@Al_2O_3$	650	98400	1.6wt% Ni	3.0×10^{-4}	[4]
Ni/La_2O_3-LOC	700	300000	5.7wt%Ni	2.1×10^{-5}	[5]
Ru/CeO_2			0.5wt%Ru	3.4×10^{-4}	
Ru/TiO_2	700	180000	0.5wt%Ru	1.6×10^{-4}	[6]
Ru/Al_2O_3			0.5wt%Ru	7.0×10^{-5}	
PdNi-MgO	700	70000	0.2wt%Pd 2.0wt%Ni	5.6×10^{-5}	[7]
ALD Ni/γ-Al_2O_3	850	375000	4wt%Ni	8.6×10^{-4}	[8]
1.5CeO_2-x-NSNT	750	96000	16wt%Ni	2.5×10^{-4}	[9]

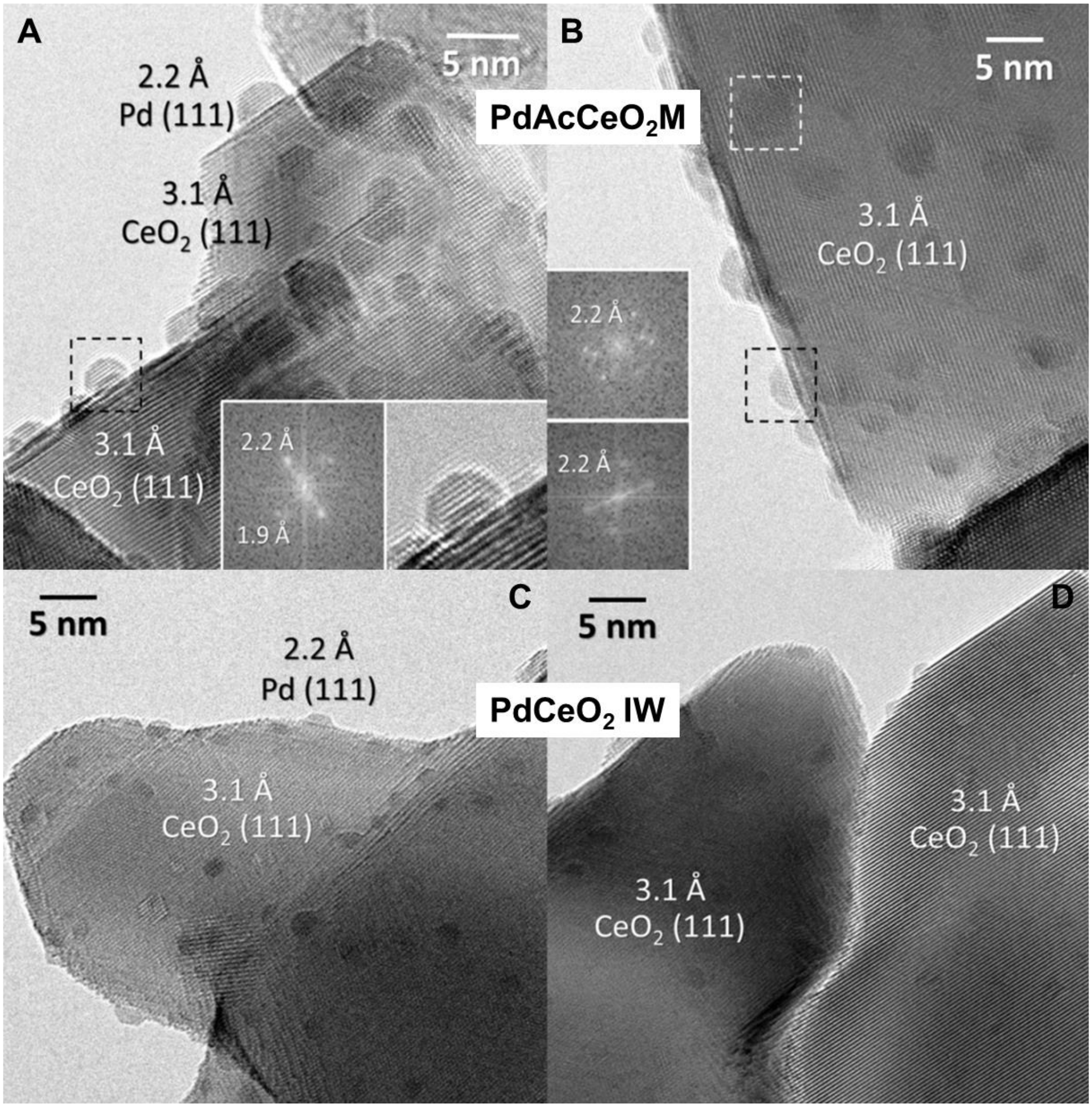

Figure S5. HRTEM images of (A-B) $PdAcCeO_2M$ after H_2 reduction treatment at 400 °C. Insets show the Fourier Transform corresponding to the respective image inside the rectangles under high magnification. (C-D) images of $PdCeO_2IW$ after H_2 reduction treatment at 400 °C.

Table S2. Pd K-edge EXAFS Fitting Results.

Sample	Shell	*R* (Å)	C.N.	σ^2(Å^2)	ΔE_o(eV)
$PdAcCeO_2M$	Pd-Pd	2.837 ± 0.003	10.4 ± 0.6	0.007	-7.5
$PdCeO_2IW$	Pd-Pd	2.785 ± 0.003	10.7 ± 0.6	0.006	-8.6

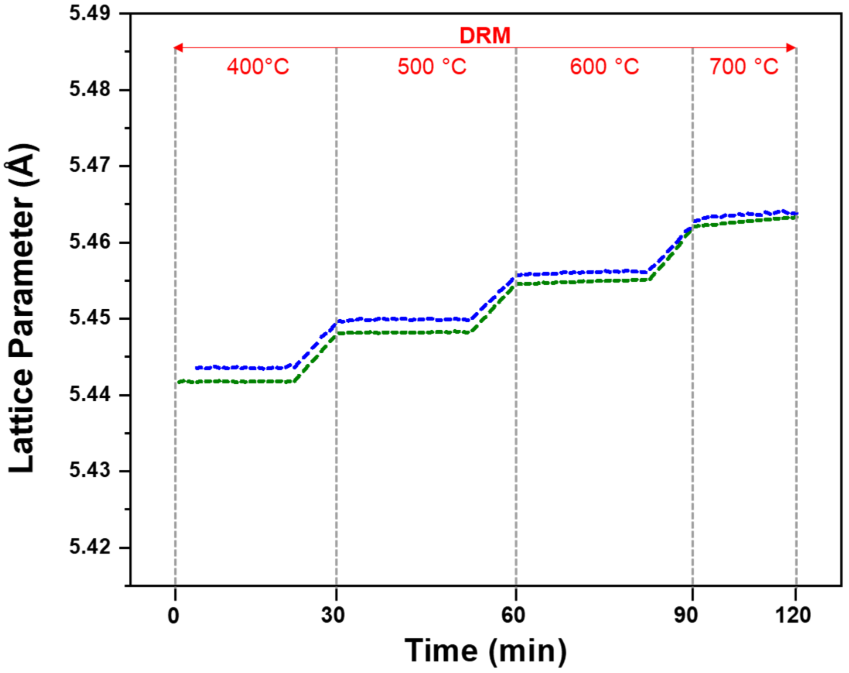

Figure S6. Representative Rietveld refinement of the ceria lattice parameter of $PdAcCeO_2M$ (green) and $PdCeO_2IW$ (blue) under DRM conditions. Reaction conditions: 25%CO_2/25%CH_4/50%He mixture.

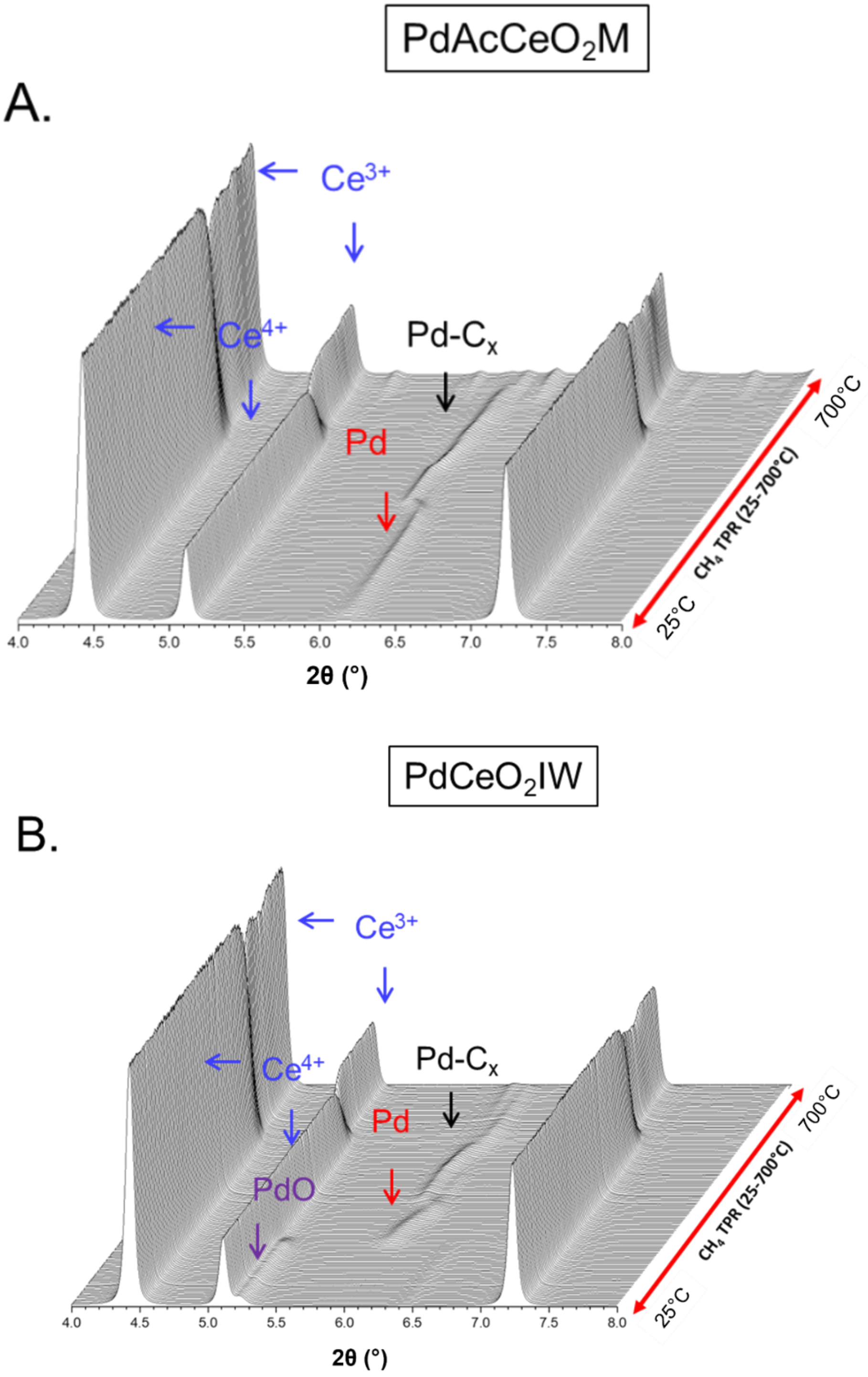

Figure S7. *In-situ* XRD profiles of (A) $PdAcCeO_2M$ and (B) $PdCeO_2IW$ samples in a CH_4 atmosphere. Reaction conditions: 5 mL/min CH_4 + 5 mL/min He, ramping to 700 °C with a 5 °C/min ramping rate.

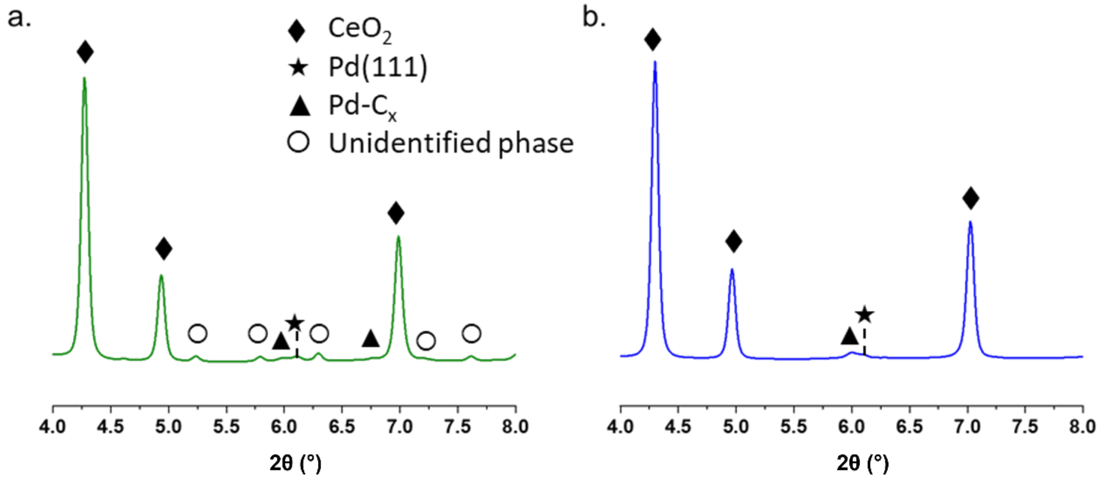

Figure S8. *In-situ* XRD profiles at 700 °C of (a) $PdAcCeO_2M$ and (b) $PdCeO_2IW$ samples in a CH_4 atmosphere.

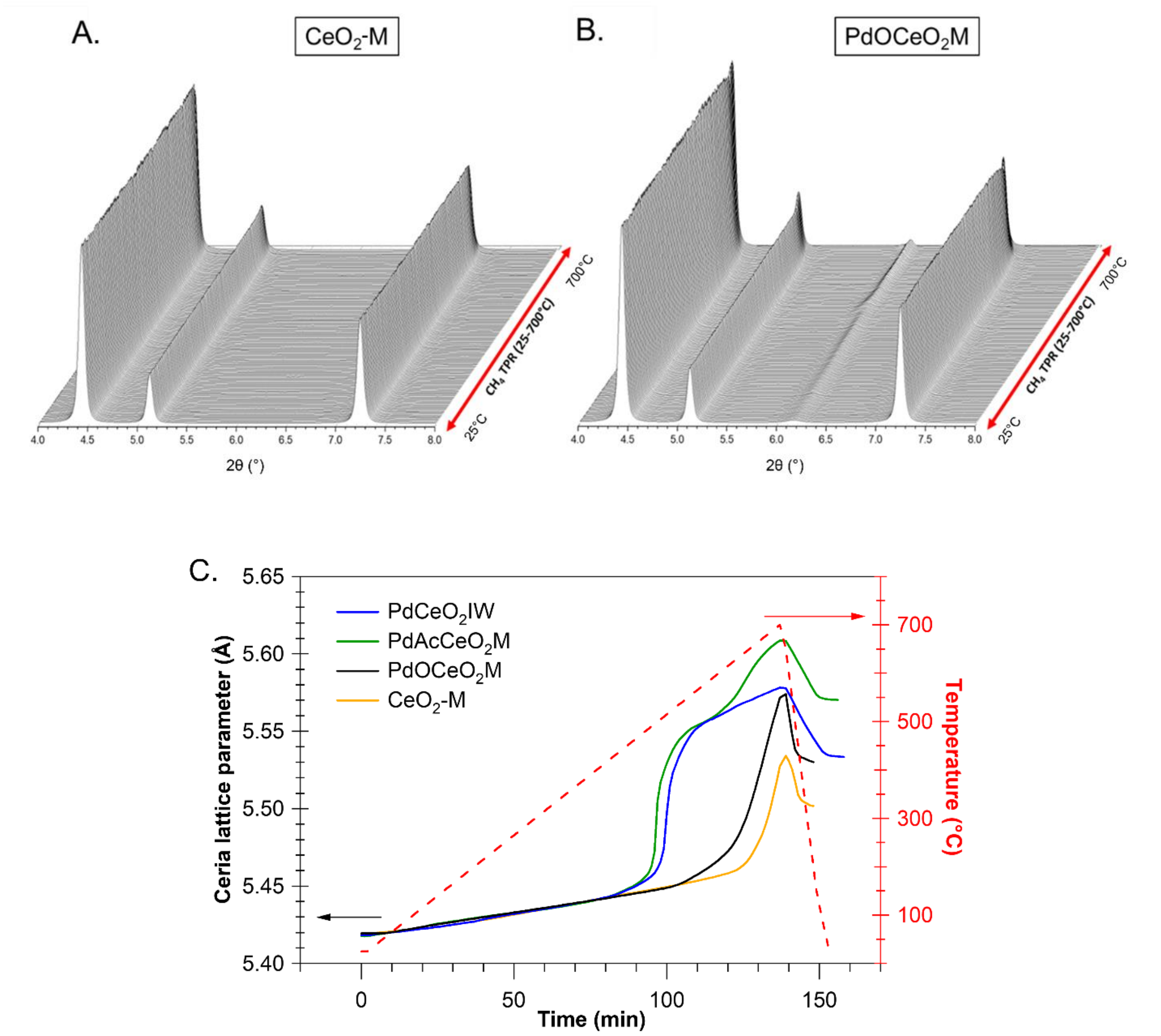

Figure S9. *In-situ* XRD profiles of (A) CeO_2-M and (B) $PdOCeO_2M$ samples in a CH_4 atmosphere. Reaction conditions: 5 mL/min CH_4 + 5 mL/min He, ramping to 700 °C with a 5 °C/min ramping rate. (C) Rietveld refinement of ceria lattice parameters of $PdAcCeO_2M$, $PdCeO_2IW$, $PdOCeO_2M$ and CeO_2-M under *in-situ* CH_4-TPR XRD experiments.

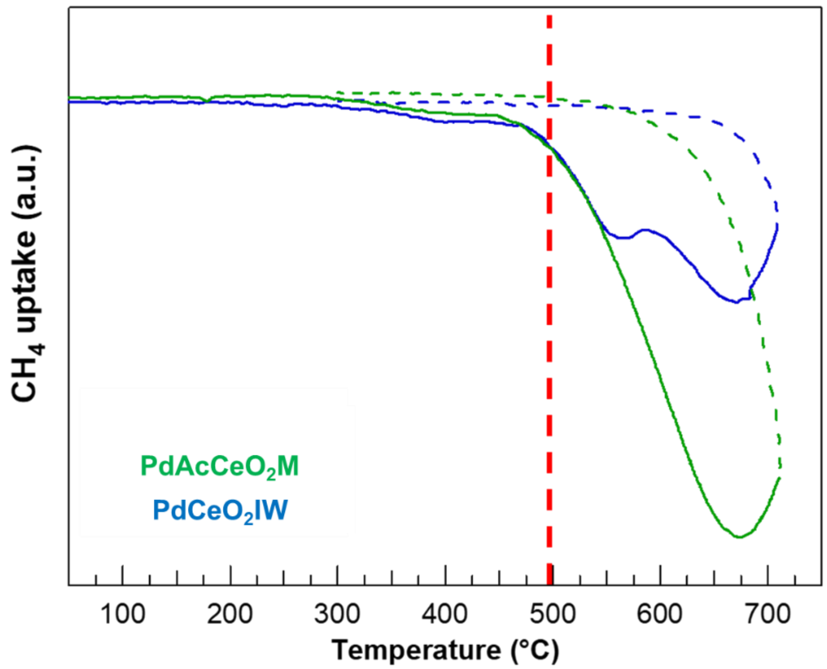

Figure S10. CH_4-TPR uptake measurements under a 25%CH_4/He atmosphere at a GHSV ≈ 400’000 h^{-1}.

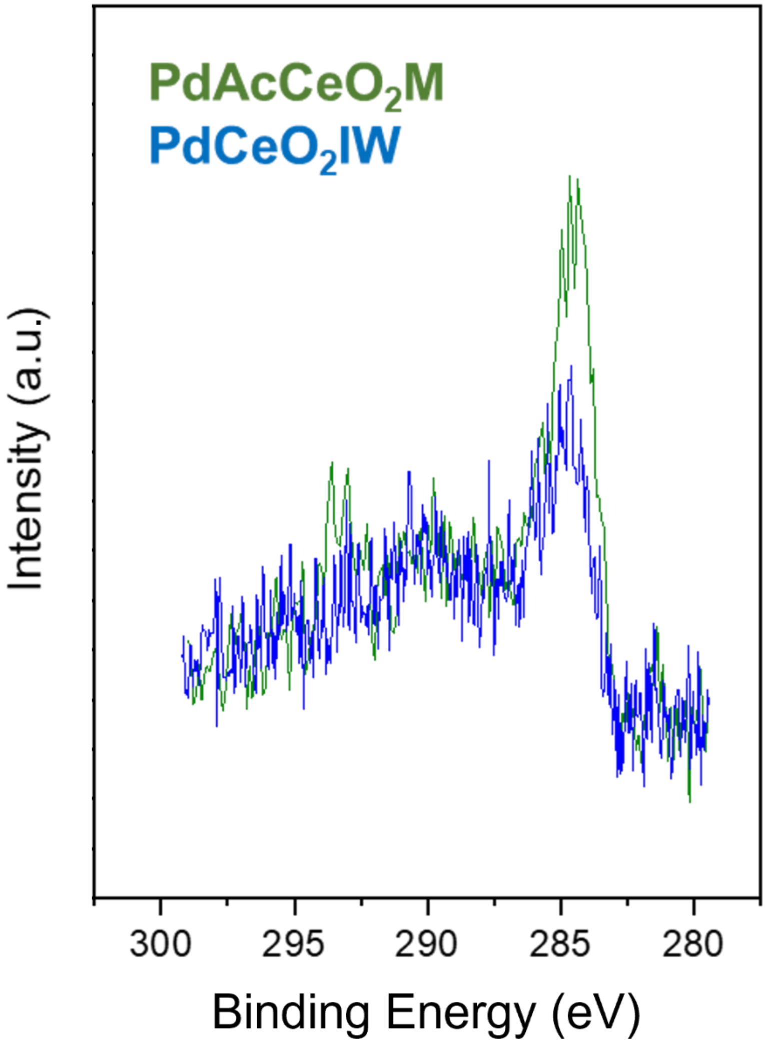

Figure S11. *In-situ* XPS profiles at 500 °C of (a) PdAcCeO_2M and (b) PdCeO_2IW samples under 10 mTorr of CH_4.

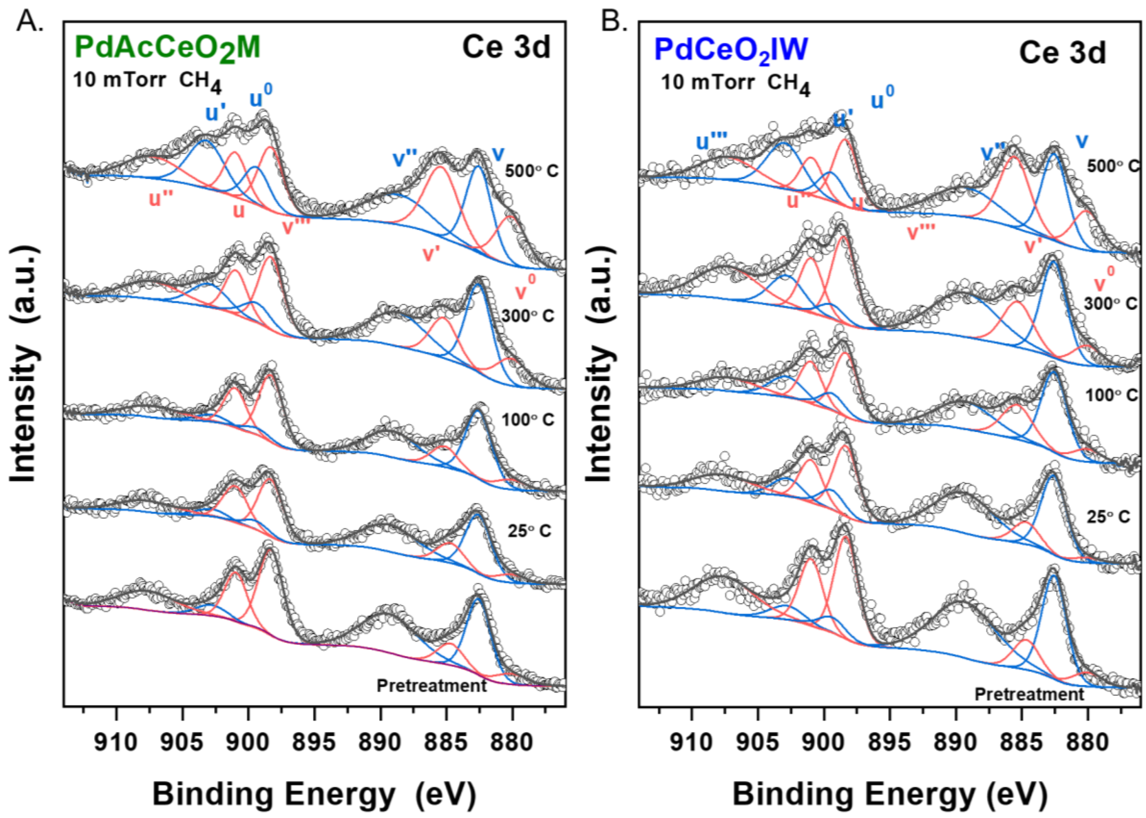

Figure S12. Ce 3d AP-XPS spectra of (A) $PdAcCeO_2M$ and (B) $PdCeO_2IW$ samples in a 10 mTorr CH_4 atmosphere, from 25 to 500 °C.

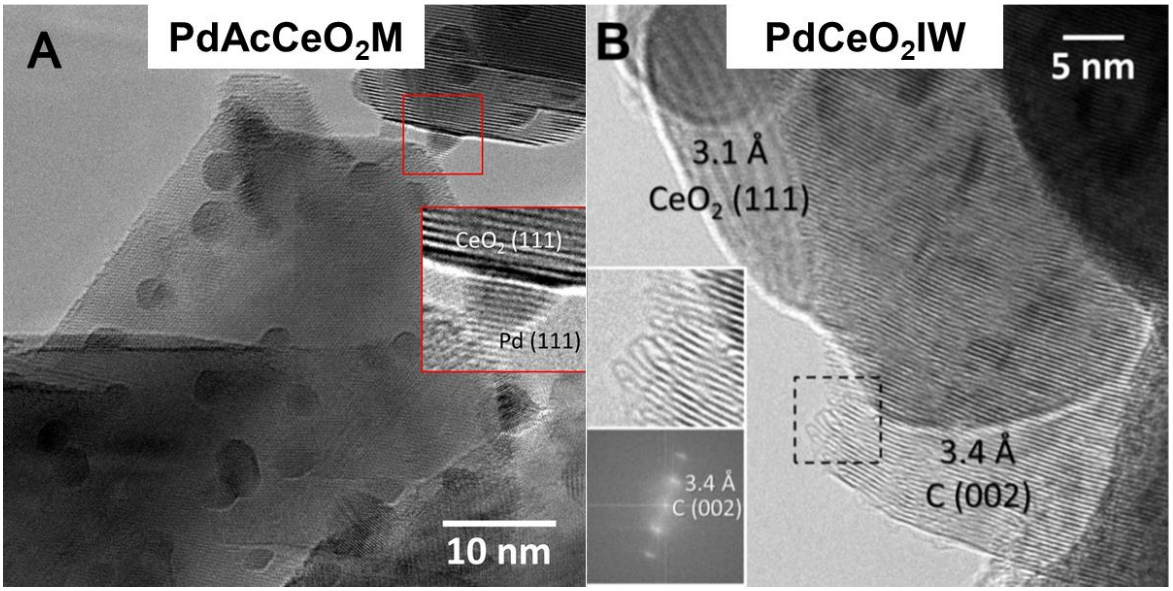

Figure S13. HRTEM images of (A) $PdAcCeO_2M$ and (B) $PdCeO_2IW$ after 24 h at 700 °C under DRM conditions.

Table S3. Particle size obtained through HR-TEM measurements under different pre-treatment and/or reaction conditions.

Treatment	$PdAcCeO_2M$ (nm)	$PdCeO_2IW$ (nm)
H_2 – 400 °C	3.5	2.0
DRM – 400 °C	3.8	2.8
DRM – 700 °C	3.3	2.4
H_2 – 700 °C	5.7	5.0
He – 700 °C	5.2	4.6

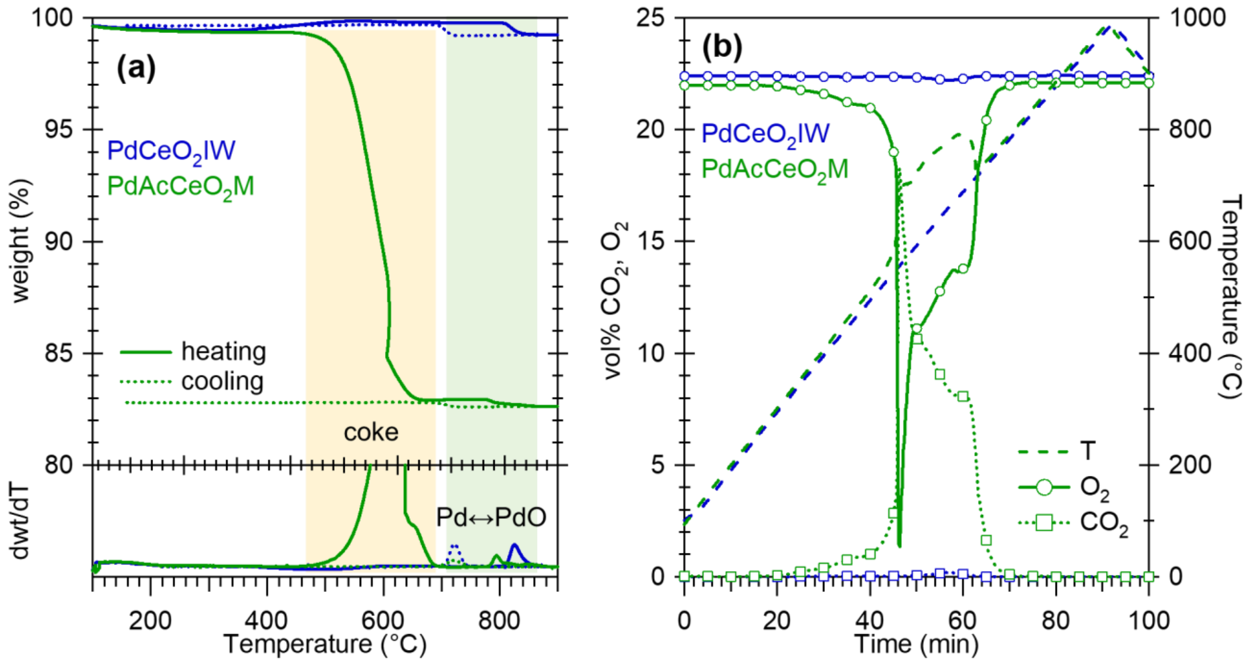

Figure S14. (a) Thermogravimetric Analysis (TGA) and **(b)** Temperature Programmed Oxidation (TPO) tests of the $PdCeO_2IW$ and $PdAcCeO_2M$ samples after prolonged stability test under DRM conditions (24 h, 700 °C). TGA experiments were carried out in a Q500 (TA Instruments) exposing ca. 15 mg of sample loaded in a platinum pan to a flow of air (60 mL/min) and heating to 900 °C at 10 °C/min while continuously monitoring its weight evolution. Oxidation tests were performed loading 60 mg of sample in a quartz tube reactor on a quartz wool bed; the catalyst powder was exposed to a flow of air (60 mL/min) and heated at 10 °C/min to 1000 °C, while the O_2 and CO_2 gas concentration were continuously measured with an on line ABB Magnos 106 – Uras 14 gas analyzer.

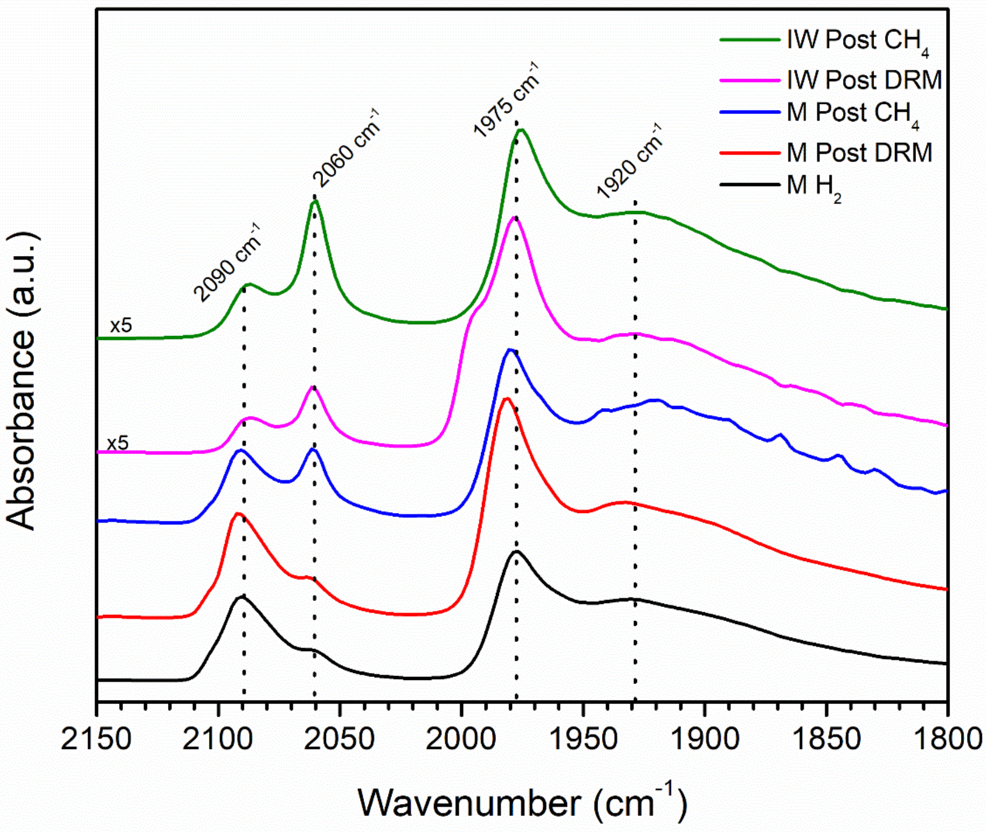

Figure S15. *In-situ* DRIFTS spectra collected over $PdAcCeO_2M$ (M) and $PdCeO_2IW$ (IW) after exposure to 10%CO/He for 15 min and a subsequent He purge for 5 min, at room temperature. All samples were pretreated under H_2 at 400 °C for 1 h and DRM indicated samples were treated to an additional hour under DRM conditions (CH_4/CO_2/He = 5/5/30 mL/min) at 400 °C while CH_4 indicated spectra were treated under a CH_4 TPR (CH_4/He 12.5/37.5 mL/min) to 400 °C using a ramp rate of 10 °C/min and held at 400 °C for 5 min to stabilize the temperature. All spectra were collected at room temperature.

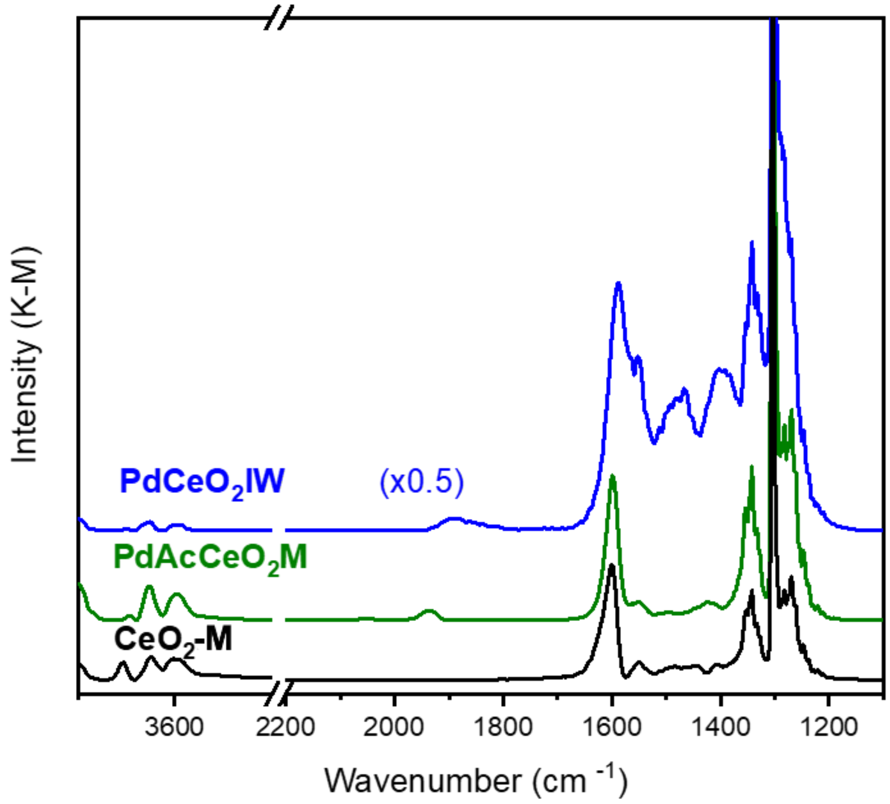

Figure S16. *In-situ* DRIFTS spectra collected over $PdAcCeO_2M$, $PdCeO_2IW$ and CeO_2-M under steady-state conditions (CH_4/CO_2/He = 5/5/30 mL/min) at 250 °C.

Table S4. Assignment of IR bands.

Wavenumber (cm^{-1})	Assignment	Reference
2350	$CO_{2,gas}$	[10]
1305	$CH_{4,gas}$	[11, 6]
2085	CO-O_v-PdO(101)	[12]
2180-2130	CO-Pd^{2+} linear	[13]
2130-2100	CO-Pd^{+} linear	[13-15]
2130-2100	CO-$Pd^{\delta+}$(C) linear	[16]
2090-2034	CO-Pd^{0} linear	[10,12, 17-19]
1980-1890	CO-Pd^{0} bridged	[10, 20]
1900-1830	CO-Pd^{0} hollow	[18]
1577-1519,1353	Carbonates	[10, 20]
1569-1600	Formates	[20]

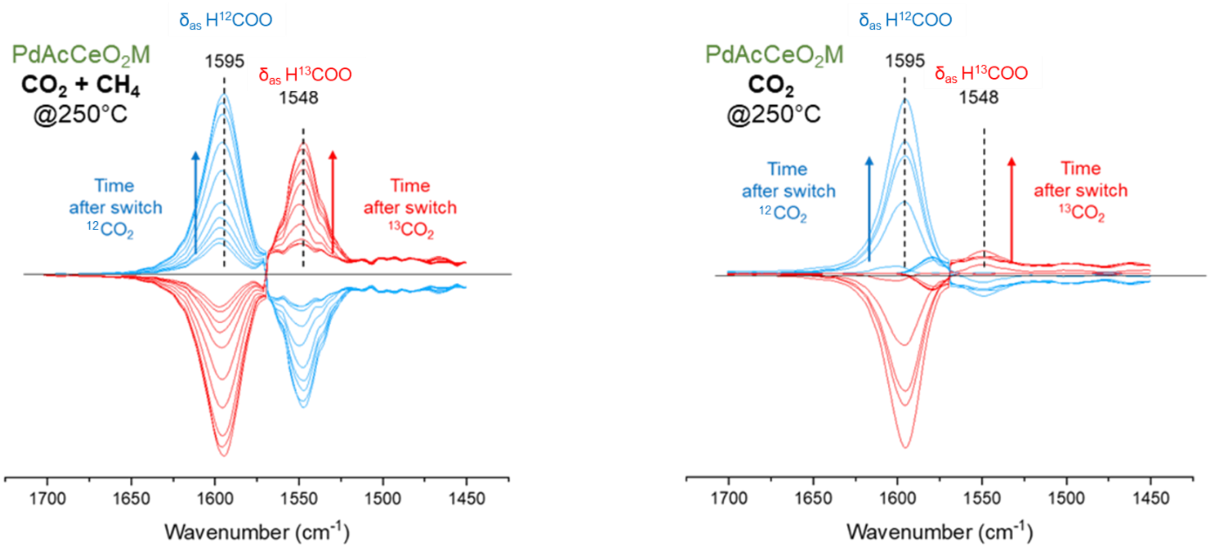

Figure S17. Time-resolved *in-situ* FTIR difference spectra of surface species formed during switch from $^{12}CO_2$ to $^{13}CO_2$ (red spectra) and $^{13}CO_2$ to $^{12}CO_2$ (blue spectra) mixture at 250°C at 1 atm under CO_2 and CH_4 (left) ($CH_4/^{12}CO_2$ ($^{13}CO_2$) /He = 5/5/30 mL/min) and only CO_2 (right) ($^{12}CO_2$ ($^{13}CO_2$) /He = 5/35 mL/min at 250 °C). Total time was from 0 to 100 seconds and each scan was collected every 5 seconds.

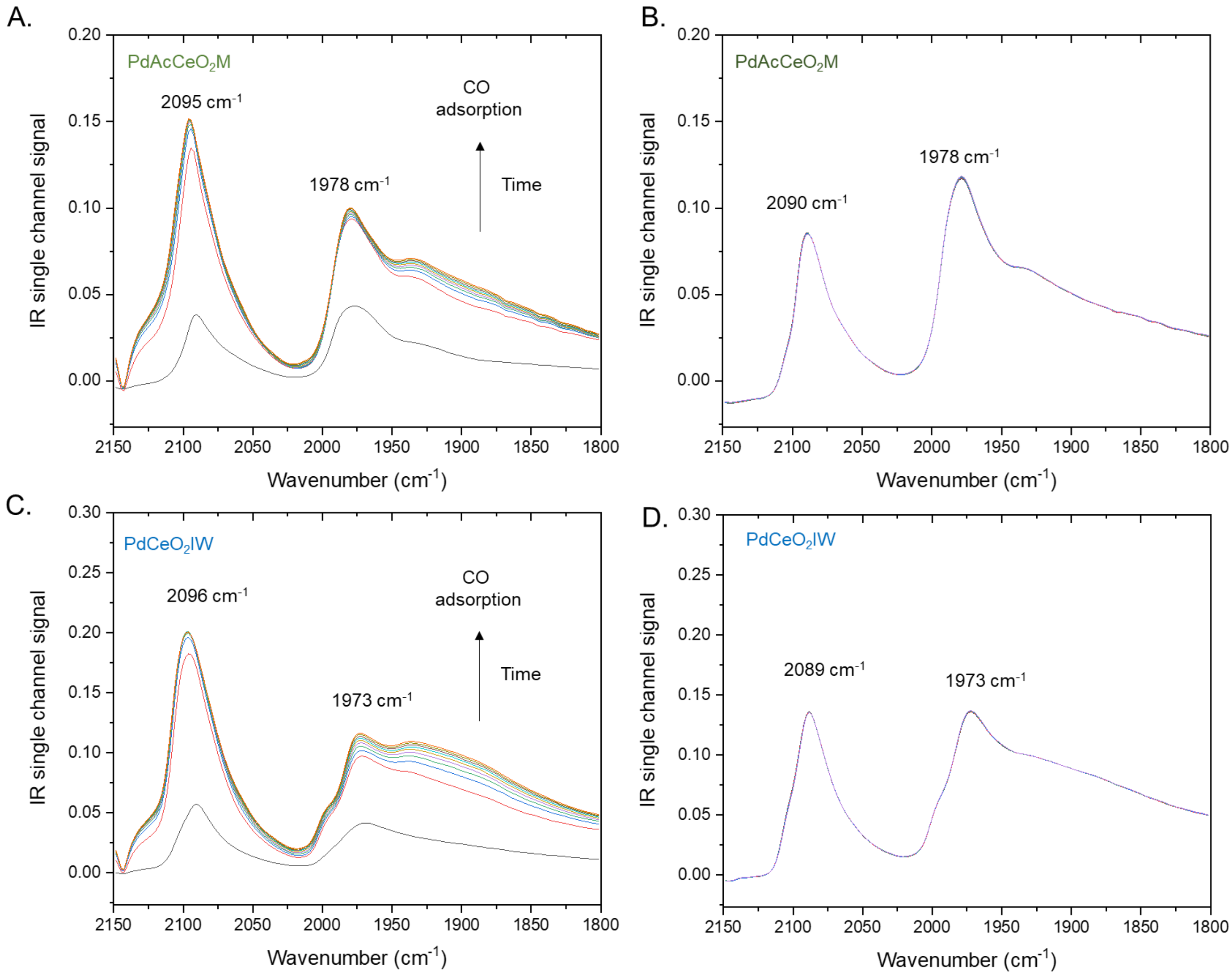

Figure S18. (A) *In-situ* DRIFTS spectra collected at room temperature on $PdAcCeO_2M$ under CO and (B) subsequent He purge. (C) $PdCeO_2IW$ under CO and (D) subsequent He purge. Conditions: 50 mL/min flow (UHP He for purge or 10 % CO in balance He).

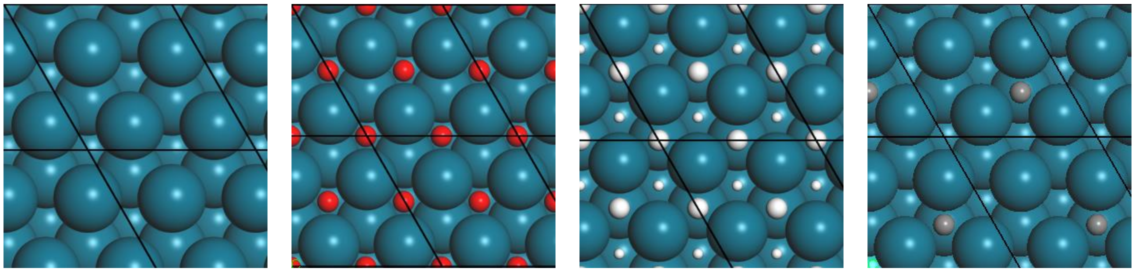

a **b** **c** **d**

Figure S19. Top view of DFT-optimized surfaces: a: Pd(111), b: Pd(111)$_{O\text{-}1ML}$, c: PdH(111), d: Pd(111)$_{C\text{-}1ML}$. Atom colors: Pd: blue, H: white, O: red, C: grey.

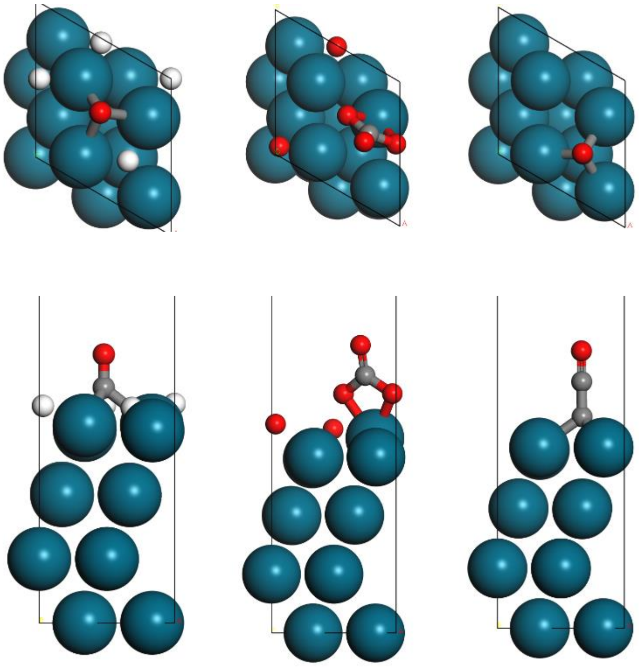

a **b** **c**

Figure S20. Top and side view of DFT-optimized *CO adsorption on Pd_3-fcc site of Pd(111)$_{H\text{-}1ML}$ (a), O_2-bridge site of Pd(111)$_{O\text{-}1ML}$ (b) and C-top site of Pd(111)$_{C\text{-}0.25ML}$ (c). Atom colors: Pd: blue, H: white, O: red, C: grey.

References

1. Reid, R.; Prausnitz, J.; Poling, B., Diffusion coefficients. *The properties of gases and liquids* **1987**.

2. Davis, M. E.; Davis, R. J., *Fundamentals of chemical reaction engineering*. Courier Corporation: 2012.

3. Pino, L.; Italiano, C.; Laganà, M.; Vita, A.; Recupero, V., Kinetic study of the methane dry (CO_2) reforming reaction over the $Ce_{0.70}La_{0.20}Ni_{0.10}O_{2-\delta}$ catalyst. *Catal. Sci. Technol.* **2020,** *10* (8), 2652-2662.

4. Margossian, T.; Larmier, K.; Kim, S. M.; Krumeich, F.; Fedorov, A.; Chen, P.; Müller, C. R.; Copéret, C., Molecularly Tailored Nickel Precursor and Support Yield a Stable Methane Dry Reforming Catalyst with Superior Metal Utilization. *J. Am. Chem. Soc.* **2017,** *139* (20), 6919-6927.

5. Li, X.; Li, D.; Tian, H.; Zeng, L.; Zhao, Z.-J.; Gong, J., Dry reforming of methane over Ni/La_2O_3 nanorod catalysts with stabilized Ni nanoparticles. *Appl. Catal., B* **2017,** *202*, 683-694.

6. Liu, Z.; Zhang, F.; Rui, N.; Li, X.; Lin, L.; Betancourt, L. E.; Su, D.; Xu, W.; Cen, J.; Attenkofer, K.; Idriss, H.; Rodriguez, J. A.; Senanayake, S. D., Highly Active Ceria-Supported Ru Catalyst for the Dry Reforming of Methane: In Situ Identification of $Ru^{\delta+}$–Ce^{3+} Interactions for Enhanced Conversion. *ACS Catal.* **2019,** *9* (4), 3349-3359.

7. Singha, R.; Shukla, A.; Sandupatla, A.; Deo, G.; Bal, R., Synthesis and catalytic activity of a Pd doped Ni–MgO catalyst for dry reforming of methane. *J. Mater. Chem. A* **2017,** *5* (30), 15688-15699.

8. Shang, Z.; Li, S.; Li, L.; Liu, G.; Liang, X., Highly active and stable alumina supported nickel nanoparticle catalysts for dry reforming of methane. *Appl. Catal., B* **2017,** *201*, 302-309.

9. Guo, D.; Lu, Y.; Ruan, Y.; Zhao, Y.; Zhao, Y.; Wang, S.; Ma, X., Effects of extrinsic defects originating from the interfacial reaction of CeO_{2-x}-nickel silicate on catalytic performance in methane dry reforming. *Appl. Catal., B* **2020,** *277*, 119278.

10. Demoulin, O.; Navez, M.; Ruiz, P., Investigation of the behaviour of a Pd/γ-Al_2O_3 catalyst during methane combustion reaction using in situ DRIFT spectroscopy. *Appl. Catal., A* **2005,** *295* (1), 59-70.

11. Stotz, H.; Maier, L.; Boubnov, A.; Gremminger, A.; Grunwaldt, J.-D.; Deutschmann, O., Surface reaction kinetics of methane oxidation over PdO. *J. Catal.* **2019,** *370*, 152-175.

12. Martin, N. M.; Van den Bossche, M.; Grönbeck, H.; Hakanoglu, C.; Zhang, F.; Li, T.; Gustafson, J.; Weaver, J. F.; Lundgren, E., CO Adsorption on Clean and Oxidized Pd(111). *J. Phys. Chem. C* **2014,** *118* (2), 1118-1128.

13. Kyriakidou, E. A.; Lee, J.; Choi, J.-S.; Lance, M.; Toops, T. J., A comparative study of silver- and palladium-exchanged zeolites in propylene and nitrogen oxide adsorption and desorption for cold-start applications. *Catal. Today* **2021,** *360*, 220-233.

14. Terekhina, O.; Roduner, E., FTIR Spectroscopic Investigation of Zeolite-Supported Pd–Ag Bimetallic Clusters. *J. Phys. Chem. C* **2012,** *116* (12), 6973-6979.

15. Sheu, L.; Knözinger, H.; Sachtler, W., Ship-in-a-bottle formation of $Pd_{13}(CO)_x$ clusters in zeolite NaY. *Catal. Lett.* **1989,** *2* (3), 129-137.

16. Jones, W.; Wells, P. P.; Gibson, E. K.; Chutia, A.; Silverwood, I. P.; Catlow, C. R. A.; Bowker, M., Carbidisation of Pd Nanoparticles by Ethene Decomposition with Methane Production. *ChemCatChem* **2019,** *11* (17), 4334-4339.

17. Lear, T.; Marshall, R.; Antonio Lopez-Sanchez, J.; Jackson, S. D.; Klapötke, T. M.; Bäumer, M.; Rupprechter, G.; Freund, H.-J.; Lennon, D., The application of infrared spectroscopy to probe the surface morphology of alumina-supported palladium catalysts. *J. Chem. Phys.* **2005,** *123* (17), 174706.

18. Khivantsev, K.; Jaegers, N. R.; Koleva, I. Z.; Aleksandrov, H. A.; Kovarik, L.; Engelhard, M.; Gao, F.; Wang, Y.; Vayssilov, G. N.; Szanyi, J., Stabilization of super electrophilic Pd^{+2} cations in small-pore SSZ-13 zeolite. *J. Phys. Chem. C* **2019,** *124* (1), 309-321.

19. Jbir, I.; Couble, J.; Khaddar-Zine, S.; Ksibi, Z.; Meunier, F. d.; Bianchi, D., Individual heat of adsorption of adsorbed CO species on palladium and Pd–Sn nanoparticles supported on Al_2O_3 by using temperature-programmed adsorption equilibrium methods. *ACS Catal.* **2016,** *6* (4), 2545-2558.

20. Li, C.; Sakata, Y.; Arai, T.; Domen, K.; Maruya, K.-i.; Onishi, T., Carbon monoxide and carbon dioxide adsorption on cerium oxide studied by Fourier-transform infrared spectroscopy. Part 1.-Formation of carbonate species on dehydroxylated CeO_2, at room temperature. *J. Chem. Soc., Faraday Trans. 1* **1989,** *85* (4), 929-943.
